# Transcriptomic Profiling of Zebrafish Mutant for *cdkl5* Reveals Dysregulated Gene Expression Associated with Neuronal, Muscle, Visual and Skeletal Development

**DOI:** 10.3390/ijms26136069

**Published:** 2025-06-24

**Authors:** Tatiana Varela, Débora Varela, Natércia Conceição, M. Leonor Cancela

**Affiliations:** 1 Centre of Marine Sciences, University of Algarve, 8005-139 Faro, Portugal; a44770@ualg.pt (T.V.); a44771@ualg.pt (D.V.); 2 Faculty of Medicine and Biomedical Sciences, University of Algarve, 8005-139 Faro, Portugal; 3 Algarve Biomedical Center, University of Algarve, 8005-139 Faro, Portugal

**Keywords:** zebrafish model, Cdkl5, RNA-seq, transcriptomic, CDKL5 deficiency disorder

## Abstract

Zebrafish is a well-recognized model for studying human genetic disorders. Recently, we proposed the homozygous *cdkl5^sa21938^* mutant zebrafish as a model of CDKL5 deficiency disorder (CDD), a developmental epileptic encephalopathy with diverse symptoms. This study aimed to explore Cdkl5-associated molecular mechanisms in zebrafish and assess their similarity to those in mammals. We conducted RNA sequencing on whole *cdkl5*^−/−^ zebrafish and wild-type siblings at 5 and 35 days post-fertilization (dpf) to compare their gene expression profiles. Most significant differentially expressed genes (DEGs) were related to muscle, neuronal, and visual systems which are affected in CDD. Gene Ontology analysis revealed downregulated DEGs enriched in muscle development, extracellular matrix, and actin cytoskeleton functions at both stages, while upregulated DEGs were enriched in eye development functions at 35 dpf. The Kyoto Encyclopedia of Genes and Genomes (KEGG) analysis revealed enrichment of downregulated DEGs in focal adhesion and extracellular matrix (ECM)-receptor interaction pathways at both stages. Neuronal development DEGs were mainly downregulated at both stages, while synaptic signaling DEGs were upregulated at 35 dpf. Crossing *cdkl5*^−/−^ mutants with the Hb9:GFP transgenic line showed fewer motor neuron cells with shorter axons compared to the wild type, which may explain the impaired motor phenotype observed in zebrafish and CDD patients. Moreover, we identified key downregulated DEGs related to cartilage development at both stages and bone development at 35 dpf, potentially explaining the skeletal defects seen in zebrafish and CDD individuals. In conclusion, Cdkl5 loss in zebrafish leads to dysregulation of genes involved in CDKL5-associated functions in mammals, providing new insights into its less studied functions and phenotypes.

## 1. Introduction

Human cyclin-dependent kinase-like 5 (CDLK5) is encoded by a single-copy gene located on the short arm of the X chromosome. It is widely expressed in many tissues throughout the human body. However, it is most abundant in the brain (specifically in the forebrain, cerebral cortex, hippocampus, striatum, and olfactory bulb), testicles, and thymus [1,2,3]. During the embryonic stages, *CDKL5* expression is minimal but it quickly increases in postnatal stages and throughout neuronal development and synaptogenesis, indicating that CDKL5 is crucial for early normal brain development and function [3,4,5]. At a subcellular level, it is found both in the nucleus and in the cytoplasm, where it plays specific roles involved in important neuronal processes [6].

CDKL5 is an important protein kinase that is associated with several essential functions through the phosphorylation of its targets and interactions or associations with other proteins [7]. Although the extent of CDKL5’s exact functions remains to be elucidated, it is known that in the cytoplasm it regulates axon outgrowth through the interaction with shootin1 (SHTN1) [8]. It is also involved in the regulation of the actin cytoskeleton and dendritic arborization through interaction with Ras-related C3 botulinum toxin substrate 1 (Rac1) [4] and in synapse formation via phosphorylation of amphiphysin 1 (AMPH1) [9,10] and the netrin G1 receptor-protein membrane (NGL-1) [5]. CDKL5 also regulates dendritic microtubule dynamics by direct phosphorylation of microtubule-associated protein 1S (MAP1S) [11,12], EB family member 2 (EB2) [12], Rho guanine nucleotide exchange factor 2 (ARHGEF2) [12] and DLG5 (Discs Large MAGUK Scaffold Protein 5) [11]. Furthermore, by direct phosphorylation of CEP131, CDKL5 also plays a role in ciliogenesis [11]. In the nucleus, CDKL5 phosphorylates the DNA methyltransferase 1 (DNMT1) [13] and the methyl-CpG-binding protein 2 (MeCP2), thus regulating gene expression through its effect on DNA methylation [14].

Disruption of CDKL5, resulting in decreased activity, leads to CDKL5 deficiency disorder (CDD), a rare and severe condition classified as a developmental epileptic encephalopathy [15,16]. Mutations in the *CDKL5* gene are responsible for the development of this disorder, which can manifest with a broad range of symptoms. It is primarily characterized by early-onset, treatment-resistant seizures (which begin within the first three months of life) and severe neurodevelopmental impairments, including deficits in motor, communication, and cognitive development [15,16,17]. Other common phenotypes include hypotonia, cortical visual impairment, microcephaly, subtle dysmorphic features, scoliosis, sleep disturbances, and gastrointestinal problems [15,18]. Currently, there is no cure available for CDD-affected individuals and since targeted therapies are scarce, treatment only focuses on seizure management and relieving the symptoms [17,18,19].

Despite extensive efforts to elucidate the molecular factors underlying the onset and progression of CDD, its development is still unclear. Several mouse models have been generated to understand how CDKL5 deficiency leads to neurological defects [20,21,22,23,24]. These models recapitulate several phenotypes of the human disorder such as abnormal eye tracking, impaired learning and memory, motor dysfunction, and changes in locomotion [20,23]. However, these studies only focus on the neurological phenotypes of the disorder; thus, our current knowledge of CDKL5 function in other organs/tissues besides the brain is still limited. The comprehension of affected gene regulation and signaling pathways is crucial for unraveling the mechanisms underlying both normal physiology and disease states. Few studies on transcriptomic analysis in the context of Cdkl5 deficiency have been reported, and those that exist were performed using specific brain regions from *Cdkl5* null mice [25]. Previously, we identified zebrafish as a promising tool to investigate CDD. We then characterized and validated the first *cdkl5* mutant zebrafish line (sa21938) [26], suggesting its potential as a valuable model for exploring the underlying mechanisms of CDD and its relevance in high-throughput drug screening [27]. This zebrafish mutant line harbors a nonsense mutation that introduces a premature stop codon, leading to either mRNA degradation or the production of a truncated protein, resulting, among other phenotypes, in altered motor behavior, increased susceptibility to seizures, and skeletal defects [27].

The main objectives of this study were to provide new insights into the molecular mechanisms and pathways linked to Cdkl5 functions with a focus on its unexplored roles and to evaluate whether the *cdkl5*^sa21938^ mutant zebrafish line shares molecular similarities with mammalian systems that reproduce the CDD phenotypes. The validation of this model would enable its use to further investigate CDD pathophysiology and contribute to the discovery of new therapeutic molecules. To that end, we investigated the transcriptomic alterations caused by the loss of Cdkl5 function in *cdkl5* homozygous zebrafish at five days post-fertilization (dpf) and 35 dpf using a high-throughput RNA sequencing approach.

## 2. Results

### 2.1. RNA-Seq Data Description and Validation

To identify direct and indirect genes and molecular pathways affected by the potential loss of Cdkl5 function, a high-throughput gene expression analysis through RNA sequencing (RNA-seq) was performed. This analysis compared the transcriptomes of *cdkl5*^−/−^ mutant zebrafish and their wild-type (WT) siblings at 5 and 35 days post-fertilization with five biological replicates in each group. The RNA-seq data was deposited in the Gene Expression Omnibus (GEO) repository (accession number: GSE294284).

A summary of the RNA-Seq descriptive data is presented in Table 1.

After sequencing, a total of 466,025,230 and 458,390,836 raw reads were generated from 5 dpf larvae and 35 dpf juveniles’ samples, respectively. The obtained sequencing error rate, the quality score of Q30, and the average GC content for all samples validate the good quality of the sequencing. Subsequent data filtering by removal of low-quality reads, adapter-containing reads, and ploy-N-containing reads from 5 dpf larvae and 35 dpf juveniles identified the high-quality clean reads, which were aligned against the zebrafish reference genome and mapping rates ranging from 87.40% to 92.41% were obtained (Table 1). The percentage of mapped reads should be higher than 70% when an adequate genome is chosen, and no contamination occurs. Therefore, these outcomes indicate that the obtained sequencing data are accurate and reliable.

### 2.2. Gene Expression Analysis of cdkl5^−/−^ Mutant and WT Siblings’ Zebrafish

**Figure 1:**
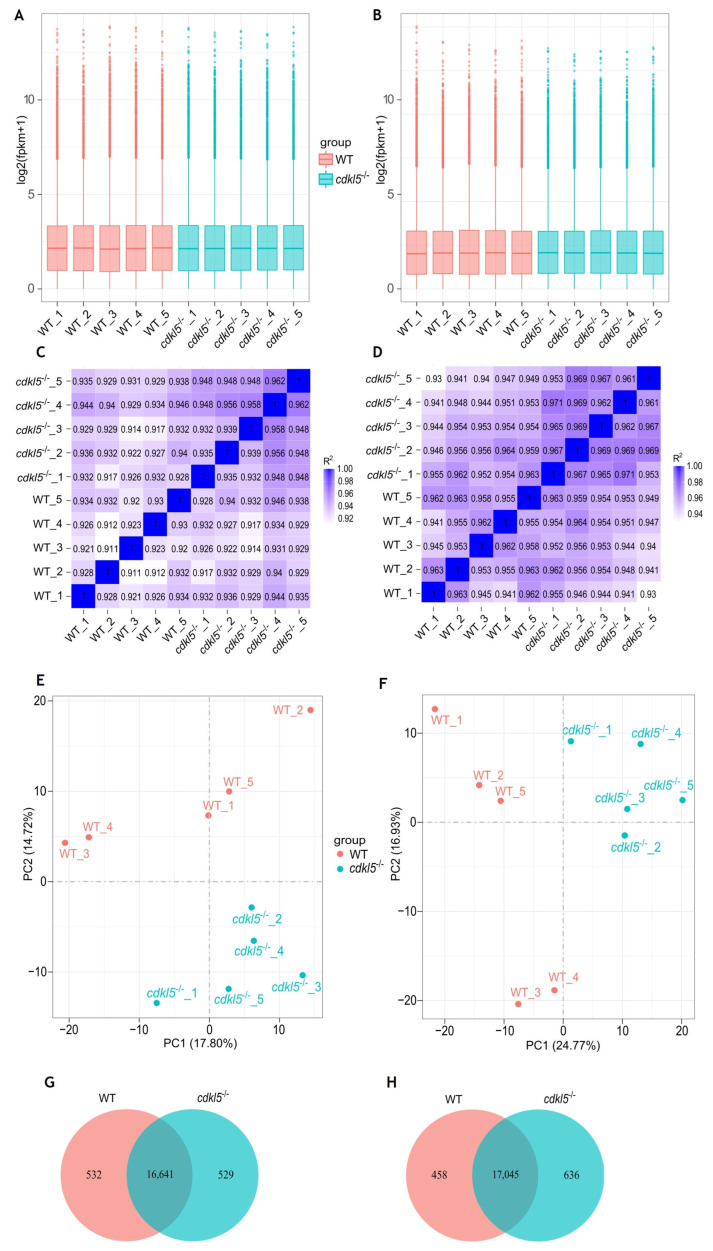
Comparison of gene expression levels between *cdkl5*^−/−^ mutant zebrafish and WT siblings at 5 dpf (**A**,**C**,**E**,**G**) and 35 dpf (**B**,**D**,**F**,**H**). (**A**,**B**) Box plots of the distribution of gene expression levels based on log2 transformed FPKM (Fragments Per Kilobase of transcript per Million mapped reads) values for each sample. The abscissa axis is the sample name, and the ordinate axis represents the log2 (FPKM + 1) values. (**C**,**D**) Heatmap of the square of Pearson correlation coefficients (R^2^) among the ten sequenced samples. (**E**,**F**) Principal Component Analysis (PCA) maps based on the gene expression value (FPKM) of samples. The abscissa axis is the first principal component (PC1) representing the most variation in the data, and the ordinate axis is the second principal component (PC2), representing the second most variation in the data. (**G**,**H**) Venn diagram of gene expression indicating the number of common and uniquely expressed genes in the WT siblings and *cdkl5*^−/−^ groups.

Expression levels for all identified genes in the *cdkl5*^−/−^ and WT samples from 5 and 35 dpf zebrafish were quantified by calculating the FPKM (Fragments Per Kilobase of transcript per Million mapped reads). The distribution of gene expression levels in the analyzed samples is indicated in the boxplot graphs of Figure 1A,B. As shown, the overall range and distribution of the FPKM values were similar and uniform across the five biological replicates of each group at both developmental ages indicating good standardization and reliability of the data.

The correlation of gene expression levels across samples is crucial for ensuring reliability and appropriate sample selection. If this is the case, the square of the Pearson correlation coefficient (R^2^) should be greater than 0.8. We performed this analysis, and the correlation coefficients (R^2^) are displayed as heatmaps in Figure 1C,D. As shown, the correlation between biological replicates within the *cdkl5*^−/−^ and WT groups was high, with R^2^ values greater than 0.911 and 0.930 for 5 and 35 dpf samples, respectively. This indicates that all data within each group were well correlated and that the biological replicates had good reproducibility.

Additionally, principal component analysis (PCA) was performed on the gene expression values (FPKM) of all samples at 5 and 35 dpf, as shown in Figure 1E,F, respectively. The similarity between samples is indicated by the distance between them, i.e., shorter distances correspond to higher similarity. Overall, in both PCA maps for 5 and 35 dpf zebrafish, the gene expression levels of samples within each group were clustered together; that is, the distance between the five biological replicates from each group was relatively small, indicating similar characteristics. In contrast, the samples from the different *cdkl5*^−/−^ and WT sibling groups were well segregated, with relatively large separation distances, indicating distinct gene expression profiles between them.

A total of 17,170 and 17,173 genes were found to be expressed in the *cdkl5*^−/−^ and WT siblings’ samples of 5 dpf zebrafish, respectively (Figure 1G). Among these genes, 16,641 were co-expressed in the two groups, 532 genes were uniquely expressed in WT siblings, and 529 genes were specifically expressed in *cdkl5*^−/−^. At 35 dpf, 17,681 and 17,503 genes were detected to be expressed in the *cdkl5*^−/−^ and WT zebrafish, respectively, with 17,045 genes being commonly expressed (Figure 1H), while 458 and 636 genes were exclusively expressed in WT siblings and in *cdkl5*^−/−^, respectively.

Altogether, these results demonstrate that the data obtained from RNA-seq in this study is both reliable and reproducible, thus suitable to be used for further bioinformatics analysis. It also indicates that the transcriptomic profile of zebrafish at different developmental stages is altered when Cdkl5 is deficient.

### 2.3. Identification of Differentially Expressed Genes Between cdkl5^−/−^ and WT Siblings’ Zebrafish

**Figure 2:**
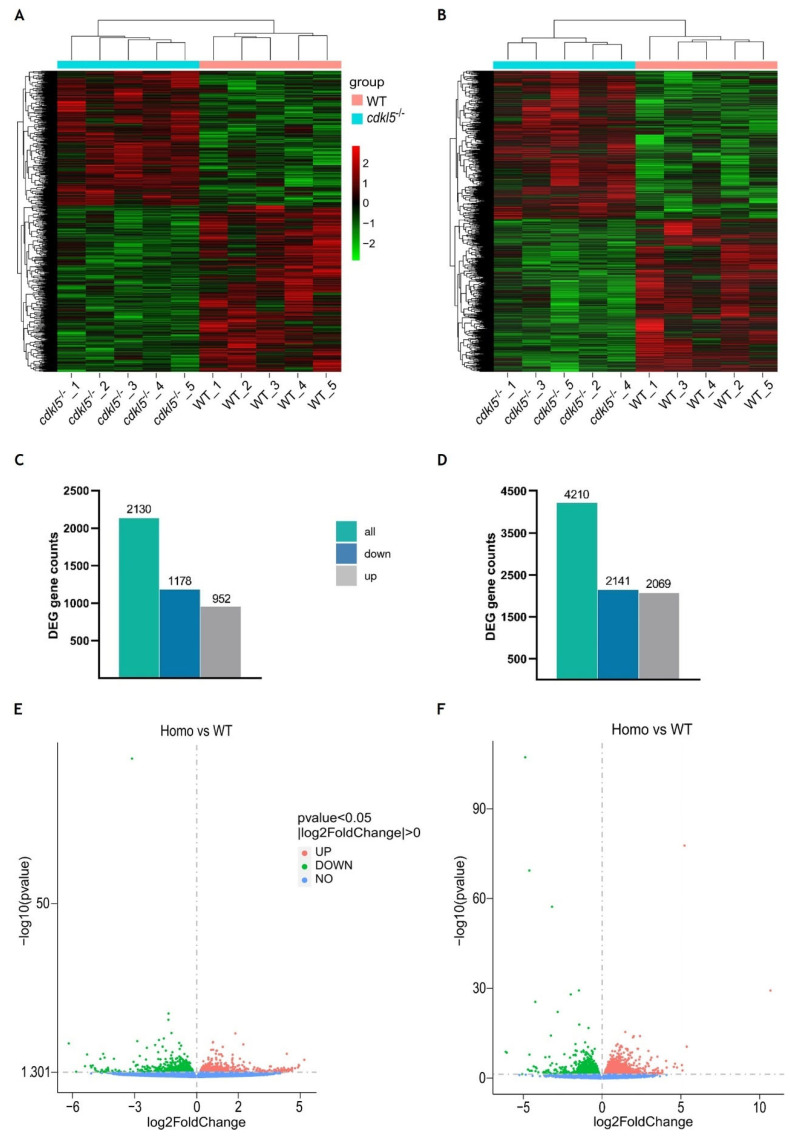
Differential gene expression analysis between *cdkl5*^−/−^ mutant zebrafish and WT siblings at 5 dpf (**A**,**C**,**E**) and 35 dpf (**B**,**D**,**F**). (**A**,**B**) Heatmap of hierarchical clustering analysis of differentially expressed genes (DEGs), based on log2(FPKM+1) values. Each column corresponds to a different sample, while each row represents a distinct gene. Red and green colors indicate genes with higher and lower expression levels, respectively. (**C**,**D**) The number of differentially expressed genes in *cdkl5*^−/−^ vs. WT groups. (**E**,**F**) Volcano plots show the distribution of the differentially expressed genes. The abscissa axis represents the value of log2FoldChange, and the ordinate axis represents the statistical significance value (−log10 (*p* value)) of gene expression between the groups. Red and green dots indicate significantly upregulated (up) and downregulated (down) genes, respectively. Blue dots represent genes with no statistically significant differences in expression (no) between the two groups.

To discover candidate genes affected by the lack of a functional Cdkl5, DESeq2 software was used to identify differentially expressed genes (DEGs) between *cdkl5*^−/−^ mutant and WT siblings’ zebrafish at both 5 and 35 dpf. A hierarchical clustering analysis based on FPKM data of all DEGs was carried out and heatmaps were generated to observe expression patterns of different genes. An evident separation between the WT siblings and *cdkl5*^−/−^ groups was observed, indicating that the *cdkl5*^−/−^ mutant fish displayed a unique gene expression pattern, showing many significant differences from the WT fish at both stages of development analyzed (Figure 2A,B).

At 5 dpf, a total of 2130 genes were found to be differently expressed between *cdkl5*^−/−^ mutant and WT larvae. Among them, 1178 genes were downregulated, and 952 genes were upregulated in the *cdkl5*^−/−^ when compared to their WT siblings (Figure 2C). At 35 dpf, a total of 4210 genes were identified as significantly dysregulated, out of which 2141 genes were downregulated and 2069 were upregulated in the *cdkl5*^−/−^ mutant juveniles in comparison to the WT (Figure 2D). The detailed information of all DEGs at 5 and 35 dpf is described in Supplementary Tables S1 and S2, respectively. Furthermore, to visualize the abundance and distribution of the differentially expressed genes between the two groups, we have generated a volcano plot for each of the developmental stages (Figure 2E,F).

Since the *cdkl5* mutant zebrafish line (sa21938) used in this work harbors a nonsense mutation that may lead to activation of the nonsense-mediated mRNA decay (NMD) mechanism, we specifically examined the expression of *cdkl5* between WT and *cdkl5*^−/−^ groups. Indeed, *cdkl5* was one of the genes found to be differentially expressed between the two groups at both stages of development. It was significantly downregulated in both 5 and 35 dpf *cdkl5*^−/−^ mutant zebrafish (Supplementary Tables S1 and S2).

The top 40 known DEGs, including the 20 most upregulated and the 20 most downregulated genes in the *cdkl5*^−/−^ mutant compared to the WT based on their significance, are listed in Table 2. Our results showed that the most significantly upregulated gene in *cdkl5*^−/−^ larvae at 5 dpf was *sort1a* (sortilin 1a), which encodes a sorting receptor or co-receptor required for the transport of several intracellular proteins from the Golgi apparatus to the cell surface of lysosomes and endosomes [28]. Ligands of sortilin include neurotensin (NTS), a neuropeptide functioning as a neurotransmitter in the brain which is encoded by the *nts* gene [29] and was also one of the most significantly upregulated genes in *cdkl5*^−/−^ mutant larvae, suggesting that Cdkl5 may be involved in the sortilin/neurotensin pathway. Additionally, *guca1c* (guanylate cyclase activator 1C) and *arr3b* (arrestin 3b) genes that have important functions in the visual phototransduction pathway [30,31] were also upregulated. The most significant downregulated gene in mutant larvae was *gamt* (guanidinoacetate N-methyltransferase), encoding for an enzyme involved in creatine synthesis and whose deficiency in humans causes a disorder characterized by developmental delay, seizures, and hypotonia [32]. Furthermore, other downregulated genes with greater significance include: *ctsl.1* and *col5a1*, involved in collagen catabolic processes and extracellular matrix organization [33,34]; *myh7ba*, involved in actin filament binding activity and microfilament motor activity being important for muscle contraction [35]; *foxj3*, involved in the regulation of muscle fiber identity and regeneration of skeletal muscle through the activation of *Mef2c* transcription [36]; *dynlt3* that encodes a member of dynein motor protein complex, involved in the transport of organelles and vesicles toward microtubule [37]; *klhdc8a*, involved in the induction of primary ciliogenesis [38]; *mbd1b*, encoding a protein involved in epigenetic regulation and its deficiency is associated with reduced neurogenesis [39]; *rs1a*, involved in the cellular organization of the retina; *lrp2b*, encoding a multifunctional receptor involved in the development of many organs such as brain and eye [40]. At 35 dpf, 9 out of the 20 most significantly upregulated genes in the *cdkl5*^−/−^ mutant juveniles were related to visual functions. These included *crygm2d18, crygm2d15*, *crygm2d16*, *crygm2d11*, *cryba2a*, *crygm2d13*, and *crygm2d14* which belong to the crystallin gene family and are involved in lens development and visual perception [41]; *rlbp1b* which encodes a retinoid-binding protein crucial for the correct function of rod and cone photoreceptors, playing several roles in the visual cycle [42]; and *lim2.4*, which encodes an eye lens protein involved in cell junction organization [43]. The two most significantly upregulated genes were *olfml3b* and *kdm5ba*. In the brain, *olfml3b* encodes a microglia-specific protein whose expression increases during neuroinflammation [44]. The gene *KDM5B* encodes a histone demethylase involved in the regulation of neuronal, bone, and muscle development [45]. Regarding the most significantly downregulated genes in 35 dpf *cdkl5*^−/−^ juveniles, several were also found to be significantly downregulated in 5 dpf *cdkl5*^−/−^ mutant larvae, including *gnl3l*, *nucks1a*, *pir*, *slc41a1*, *ccdc120*, *ugt1b5*, *dynlt3*, *myh7ba*, and *cishb*. The top two were *opn1lw1* and *opn1lw2*, which encode two red-sensitive cone opsins critical for phototransduction [46]. Other genes among the top twenty significantly downregulated included *myhc4*, *myom2a*, and *vgll2b*, three genes involved in muscle development [47,48,49]. Additionally, *vwa1*, which encodes an extracellular matrix protein essential for cartilage structure and development was downregulated; knockout of this gene in zebrafish causes cartilage dysmorphologies [50]. The gene *nfasca*, which encodes a cell adhesion protein, was also downregulated. This gene plays a key role in neurite outgrowth and fasciculation, as well as in the organization of the axon initial segment and nodes of Ranvier [51]. Mutations in this gene in humans cause a neurodevelopmental disorder with motor dysfunction [52].

### 2.4. Gene Ontology (GO) Enrichment Analysis of the Differentially Expressed Genes Between cdkl5^−/−^ and WT Zebrafish

To investigate the potential biological functions associated with the differentially expressed genes found upon *cdkl5* mutation in zebrafish, we performed Gene Ontology (GO) enrichment analysis on DEGs between *cdkl5*^−/−^ and WT at the two different stages of development analyzed. Gene Ontology is a collection of terms used to describe the functions of genes and classify gene sets. The GO terms are organized into three main categories: biological process (BP), cellular component (CC), and molecular function (MF).

**Figure 3:**
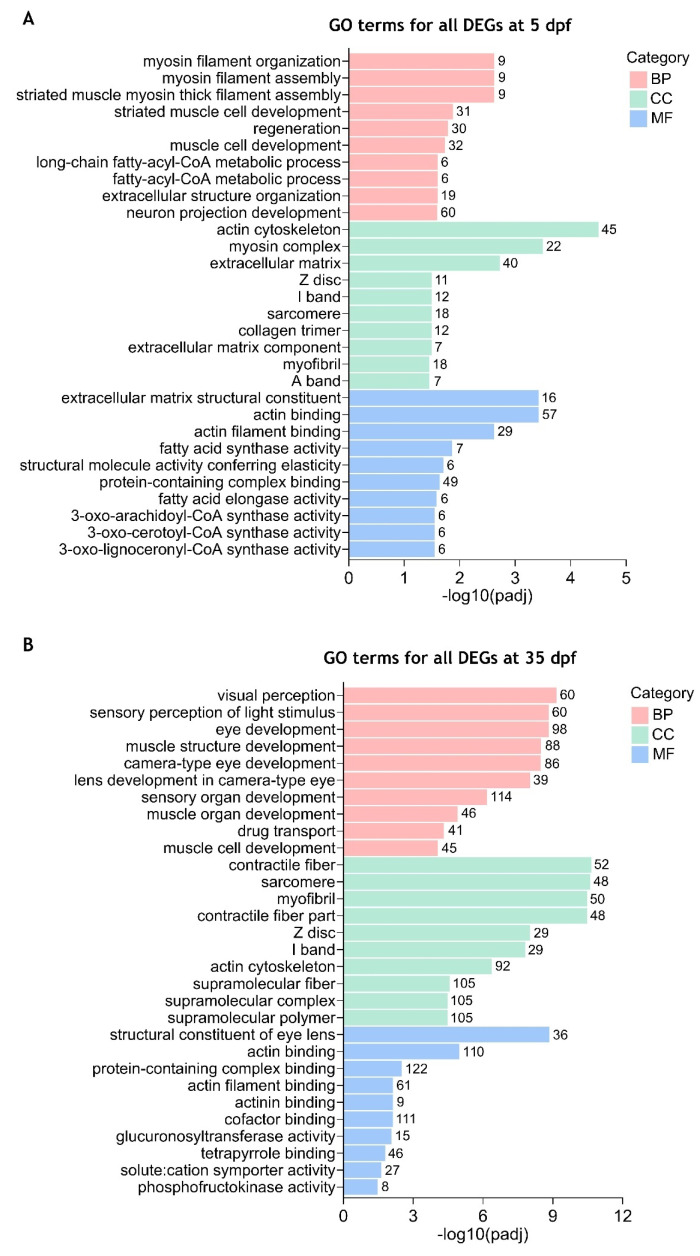
Enriched gene ontology (GO) terms for all differentially expressed genes between *cdkl5*^−/−^ mutant zebrafish and WT siblings. The top 10 significantly enriched GO terms in biological process (BP), cellular component (CC), and molecular function (MF) for all DEGs at 5 dpf (**A**) and 35 dpf (**B**) are displayed. The ordinate axis represents the GO term while the abscissa axis represents the level of significance of GO term’s enrichment, expressed as −log10(padj). Different colors represent different functional categories. The numbers above each bar indicate the number of enriched DEGs for each GO term.

By using the combined list of all 2130 DEGs found in 5 dpf mutant larvae, GO analysis revealed that the DEGs could be assigned into the three main categories and further classification resulted in the identification of several GO terms. After using a corrected *p*-value < 0.05, 65 GO terms were considered significantly enriched. Most of them were associated with biological processes (36 terms), followed by cellular components (16 terms) and molecular function (13 terms) (Supplementary Table S3). The ten most enriched GO terms of each category are shown in Figure 3A. Among these categories, the “neuron projection development” GO term belonging to the biological process contained the highest number of DEGs (60 genes). In the biological processes, the most significantly enriched GO terms included “myosin filament organization”, “myosin filament assembly” and “striated muscle myosin thick filament assembly”. The most enriched GO terms within the cellular component comprised “actin cytoskeleton”, “myosin complex” and “extracellular matrix”. Regarding the molecular function category, the most significantly enriched GO terms included “extracellular matrix structural constituent”, “actin binding” and “actin filament binding”. GO enrichment analysis of all 4210 DEGs identified in 35 dpf mutant juveniles showed that 141 GO terms were significantly enriched (padj < 0.05). As in 5 dpf mutant larvae, most of these terms were associated with biological processes (87 terms), followed by cellular components (38 terms) and molecular functions (16 terms) (Supplementary Table S4). The most significantly enriched GO terms in the BP category included “visual perception”, “sensory perception of light stimulus”, “eye development” and “muscle structure development”. Among the CC category, the top significantly enriched GO terms were “contractile fiber”, “sarcomere” and “myofibril”. In the MF category, the most significantly enriched GO terms included “structural constituent of eye lens”, “actin binding” and “protein-containing complex binding” which was also the term with the highest number of DEGs (122 genes) (Figure 3B). Furthermore, several GO terms were similarly significantly enriched in both 5 and 35 dpf *cdkl5*^−/−^ zebrafish, mainly related to muscle development, extracellular matrix organization, and actin binding. In contrast, the main alterations between 5 and 35 dpf *cdkl5*^−/−^ zebrafish were observed in categories related to the eye, which became particularly enriched at the later stage of development.

**Figure 4:**
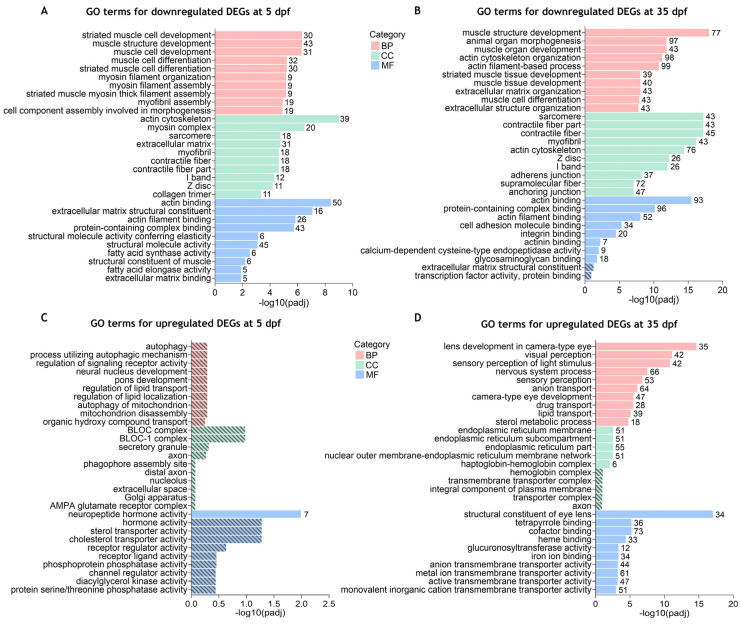
Gene ontology (GO) terms enrichment analysis for the separated downregulated and upregulated DEGs between *cdkl5*^−/−^ mutant zebrafish and WT siblings. The top 10 enriched GO terms in biological process (BP), cellular component (CC), and molecular function (MF) for the downregulated (**A**,**B**) and upregulated (**C**,**D**) DEGs at 5 dpf (**A**,**C**) and 35 dpf (**B**,**D**). Stripped bars indicate the GO terms that were not statistically significant. The numbers in front of each bar indicate the number of enriched DEGs for each GO term.

To have a better comprehension of which biological functions are being activated and repressed, we have also performed GO enrichment analysis of up- and downregulated DEGs separately. Our results showed that at 5 dpf, significantly enriched GO terms (padj < 0.05) were identified for the downregulated DEGs (Supplementary Table S5 and Figure 4A) which were similar to those identified in the combined analysis. For the upregulated DEGs, only the “neuropeptide hormone activity” GO term was found to be significantly enriched using a padj < 0.05 (Supplementary Table S6 and Figure 4B). At 35 dpf, the top significantly enriched GO terms for the downregulated DEGs were again mostly related to muscle development and contraction (skeletal and cardiac), actin cytoskeleton, and extracellular matrix (Supplementary Table S7 and Figure 4C). Additionally, GO terms such as “eye development”, “eye morphogenesis”, “central nervous system development” and “skeletal system development” were also found significantly enriched in downregulated DEGs (Supplementary Table S7). For the upregulated DEGs, the significantly enriched GO terms with higher significance were related to visual and sensory perception, lens and eye development, and nervous system processes (Supplementary Table S8 and Figure 4D). Other significantly enriched GO terms in upregulated DEGs were associated with the transport of diverse ions, activity of ion-gated channels, neurotransmitter transport and secretion, and synaptic signaling (Supplementary Table S8).

Overall, these results indicate that Cdkl5 in zebrafish is involved in important functions associated with the regulation of actin cytoskeleton, skeletal and cardiac muscle development, extracellular matrix organization, neuronal development, and eye development.

### 2.5. KEGG Pathway Enrichment Analysis of the Differentially Expressed Genes Between cdkl5^−/−^ and WT Zebrafish

The Kyoto Encyclopedia of Genes and Genomes (KEGG) enrichment analysis is used to identify biological pathways that are significantly overrepresented in a set of genes. To further elucidate the pathways affected by the *cdkl5* mutation in zebrafish, we conducted a KEGG pathway analysis on DEGs between *cdkl5*^−/−^ and WT. Pathways were considered significantly enriched when the corrected *p*-value was less than 0.05.

**Figure 5:**
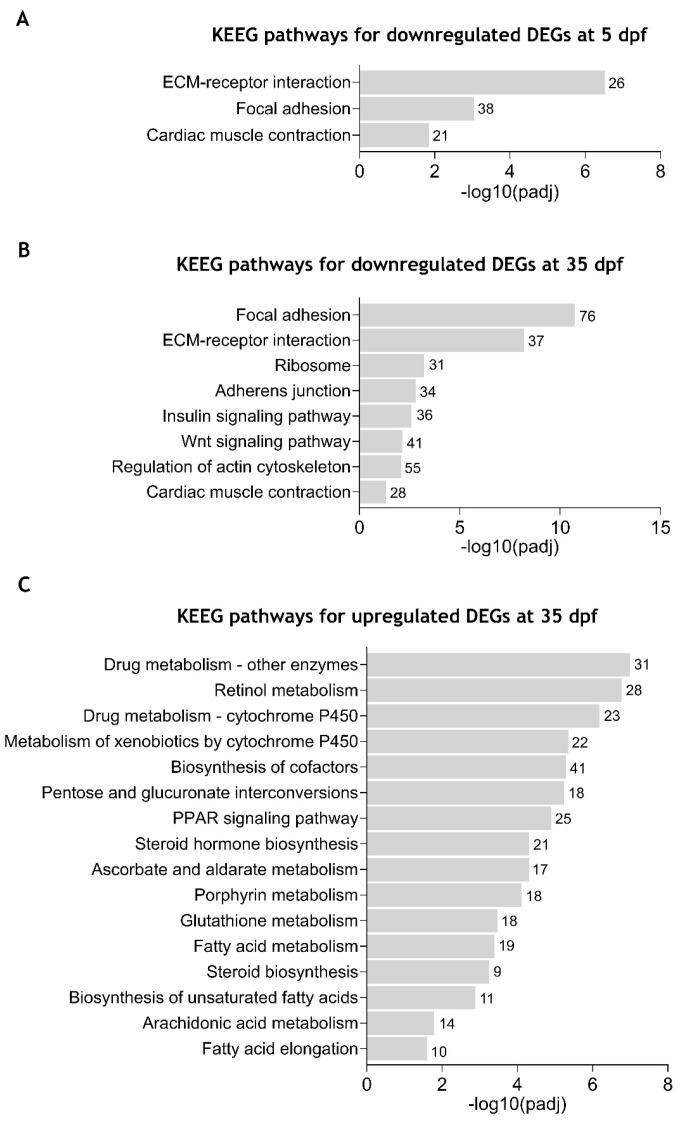
Enriched Kyoto Encyclopedia of Genes and Genomes (KEGG) pathways in zebrafish *cdkl5*^−/−^ mutants compared to WT siblings. (**A**) Significantly enriched KEGG pathways for the downregulated DEGs at 5 dpf. (**B**) Significantly enriched KEGG pathways for the downregulated DEGs at 35 dpf. (**C**) Significantly enriched KEGG pathways for the upregulated DEGs at 35 dpf. The number of DEGs contributing to each pathway is shown in front of the corresponding bar.

Separate enrichment analysis of DEGs at 5 dpf revealed three significantly enriched KEGG pathways for the downregulated DEGs, while no significantly enriched pathways were associated with upregulated DEGs. Detailed information on enriched KEGG pathways and involved genes is indicated in Supplementary Table S9. As shown in Figure 5A, downregulated DEGs were associated with “extracellular matrix (ECM)-receptor interaction” (26 DEGs), “focal adhesion” (38 DEGs), and “cardiac muscle contraction” (21 DEGs) pathways, in accordance with what was observed in the GO terms. By enrichment, analysis of downregulated DEGs at 35 dpf, eight KEEG pathways were found to be significantly enriched (Figure 5B and Supplementary Table S10). As at 5dpf, the “focal adhesion” (76 DEGs), “ECM-receptor interaction” (37 DEGs), and “cardiac muscle contraction” (28 DEGs) pathways were also enriched in the downregulated DEGs at 35 dpf. Additionally, “regulation of actin cytoskeleton” (55 DEGs), “Wnt signaling pathway” (41 DEGs), “insulin signaling pathway” (36 DEGs), “adherens junction” (34 DEGs) and “ribosome” (31 DEGs) were also identified. For the upregulated DEGs, 16 KEEG pathways were identified as significantly enriched (Figure 5C and Supplementary Table S11). Most of them were related to metabolism, including “drug metabolism-other enzymes”, “retinol metabolism”, “ascorbate and aldarate metabolism”, and “fatty acid metabolism”.

These results further demonstrate that Cdkl5 dysfunction in zebrafish at different stages of development causes downregulation of pathways essential for the interaction between cells and the extracellular matrix, thus affecting tissue organization and development.

### 2.6. Validation of the RNA-Seq Differential Gene Expression by RT-qPCR

**Figure 6:**
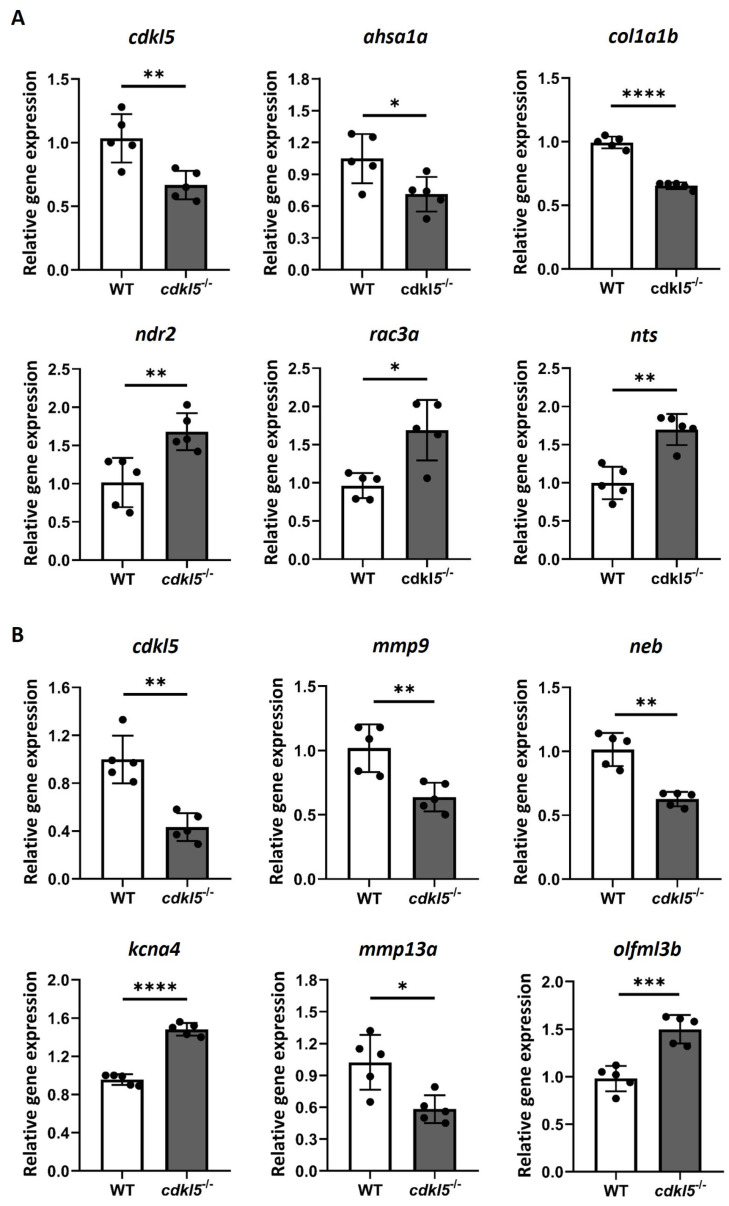
RT-qPCR analysis of a randomly selected set of DEG between *cdkl5*^−/−^ mutant zebrafish and WT siblings at 5 dpf (**A**) and 35 dpf (**B**). Relative expression values are presented as mean±SD and statistical analysis was performed using Student’s *t*-test with Welch correction. *, **, ***, and **** indicate *p* < 0.05, *p* < 0.01, *p* < 0.001 and *p* < 0.0001, respectively.

To verify the reliability and accuracy of the differentially expressed genes identified from the RNA-seq results, we randomly selected several DEGs and performed RT-qPCR (Real-time quantitative polymerase chain reaction) to assess their expression levels in *cdkl5*^−/−^ and WT zebrafish (Figure 6). In 5 dpf larvae, the expression levels of the genes *cdkl5*, *ahsa1a*, *col1a1b*, *ndr2*, *rac3a*, and *nts* were analyzed. RT-qPCR results indicated that *cdkl5*, *ahsa1a*, and *col1a1b* were downregulated, while *ndr2*, *rac3a*, and *nts* were upregulated in *cdkl5*^−/−^ larvae, consistent with the RNA-seq results. In 35 dpf juveniles, the expression levels of *cdkl5*, *mmp9*, *neb*, *kcna4*, *mmp13a*, and *olfml3b* were analyzed. Our RT-qPCR results showed a significant decrease in the expression of *cdkl5*, *mmp9*, *neb*, and *mmp13a*, while the expression of *kcna4* and *olfml3b* increased in *cdkl5*^−/−^ compared to their WT siblings, consistent with the RNA-seq results. Altogether, these findings indicate similar expression trends between RNA-seq and RT-qPCR results, therefore confirming the reliability of our RNA-seq data.

### 2.7. Impact of Cdkl5 Loss on Zebrafish Nervous System

It is widely known that CDKL5 has important roles in the brain and its deficiency in humans causes neurological defects, a main characteristic of CDKL5 deficiency disorder. To further assess the impact of *cdkl5* disruption on the nervous system of zebrafish, we specifically investigated the differentially expressed genes associated with neuronal functions between *cdkl5*^−/−^ and WT zebrafish. A subset of these DEGs is listed in Table 3.

Several DEGs related to neuronal development were found and many contributed to the enrichment of GO terms at both stages of development. At 5 dpf, GO terms such as “regulation of nervous system development”, “neuron projection development” and “axon development” were significantly enriched in a higher number of downregulated genes compared to upregulated genes. Accordingly, DEGs related to neuronal development were also mainly downregulated at 35 dpf and contributed to the enrichment of GO terms such as “central nervous system development”. Most of these genes play important roles in neuronal morphogenesis, neurite outgrowth, dendritic arborization, and axon guidance. For instance, the genes *nfasca*, *ntn1a*, *nav2b*, and *plxnb2a* were downregulated, while *stmn1b* and *pacsin1a* were upregulated at both developmental stages. Additionally, at 5 dpf, *slit1a*, *cntf*, *casp3a*, *pcdh18b*, *mycbp2* and *zc4h2* were downregulated, whereas *draxina*, *nefmb*, *nefla*, *stmn3*, *stmn4*, *srcin1b*, *amigo1*, *ndr2* and *fez1* were upregulated. At 35 dpf, genes that were downregulated included *slit2*, *pax6b*, *srgap2*, *prickle1a* and *prickle2b*. Interestingly, neuronal-related CDKL5 known targets or interactors were also identified as differentially expressed. This included the downregulated genes *iqgap1* at both developmental stages, *arhgef2*, *dlg5a*, and *rac1b* at 5 dpf and *smad3a* at 35 dpf, as well as the upregulated *shtn1* at 35 dpf (Table 3).

Moreover, genes involved in oligodendrocyte development and myelination were found to be downregulated at both development stages but only contributed to the enrichment of “glial cell development” and “oligodendrocyte development” GO terms at 35 dpf. For instance, the expression of *tfeb*, *notch3*, *prx*, *daam2*, and *plp1b* was found to be altered, in both 5 and 35 dpf *cdkl5*^−/−^ zebrafish. They were all downregulated, except for *plp1b* which was upregulated at 5 dpf and downregulated at 35 dpf. In addition, *lama2* and *actr10* were also downregulated at 35 dpf (Table 3).

Several DEGs associated with synaptic transmission were identified, with these effects being particularly pronounced at 35 dpf, where the upregulated genes significantly contributed to the enrichment of many related GO terms including “synaptic signaling”, “neurotransmitter transport”, “neurotransmitter secretion” and “synaptic vesicle exocytosis”. Genes involved in synaptic vesicle exocytosis and neurotransmission release, which is a crucial process of neurotransmission, were found mainly upregulated. Among them were *syt12*, *snap25a* and *stx12l* at both developmental stages and *syt2a*, *syt6b, syt11b*, *rab3ab*, *rph3ab*, *rimbp2*, *unc13a*, *sv2ca*, *napbb* at 35 dpf (Table 3). Additionally, several DEGs encoding ion channels playing an essential role in neuronal transmission [53,54] were dysregulated in *cdkl5*^−/−^ at both developmental stages but with a greater number identified at 35 dpf, where upregulated genes also contributed to the enrichment of GO terms related to ion channel activity. These included calcium, sodium, and potassium voltage-gated channels, which are activated by alterations in electrical membrane potential, as well as ligand-gated ion channels like GABA_A_, glutamate, glycine, and nicotinic acetylcholine receptors, activated by the neurotransmitter binding (Table 3).

Overall, these findings suggest that the *cdkl5* disruption impairs neuronal development and synaptic neurotransmission in zebrafish.

### 2.8. Effects of Cdkl5 Deficiency on Zebrafish Motor Neurons

**Figure 7:**
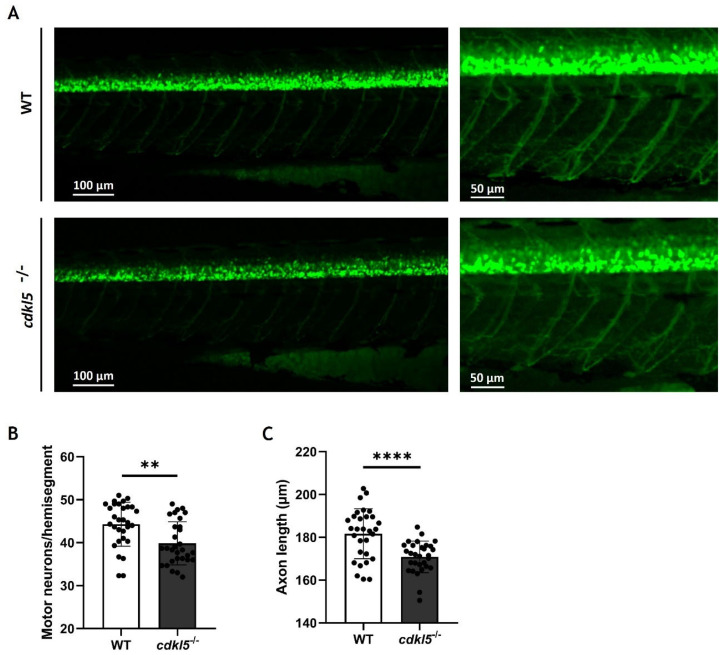
Motor neuron alterations in *cdkl5*^−/−^ zebrafish at 3 dpf. (**A**) Representative fluorescence images of the WT and *cdkl5*^−/−^ motor neurons, labeled by Tg(*hb9:GFP)*. (**B**) Number of motor neurons per hemisegment. (**C**) Axonal length of CaP motor neurons. Values are presented as mean ± SD and statistical analysis was performed using Student’s *t*-test with Welch correction. ** and **** indicate and *p* < 0.01 and *p* < 0.0001, respectively.

In our previous work, we showed that *cdkl5*^−/−^ mutant zebrafish exhibit an altered swimming performance compared to WT siblings, characterized by shorter distances traveled. Potential causes for the observed phenotype might be related to muscle weakness caused by defects in the neurons innervating the muscle. Therefore, we have investigated the development of motor neurons in the spinal cord by crossing the zebrafish mutants with the *hb9:GFP* transgenic line. The green fluorescence protein (GFP) expression is controlled by the *hb9* gene promoter, which encodes a transcription factor crucial for the differentiation of postmitotic motor neurons, thereby specifically marking primary and secondary motor neurons. Here, we analyzed the number of hb9+ motor neuron cells and the axonal projection length of the caudal primary (CaP) motor neurons at 3 dpf by fluorescence microscopy (Figure 7A). Our results showed that the average number of motor neurons (hb9+ cells) per hemisegment is significantly lower in *cdkl5*^−/−^ embryos when compared to WT siblings (Figure 7B). Additionally, the axon length of CaP motor neurons in *cdkl5*^−/−^ mutants was significantly smaller than that of WT siblings (Figure 7C).

### 2.9. Impact of cdkl5 Loss on Zebrafish Skeletal System

In our previous phenotypic analyses of the *cdkl5*^−/−^ mutant zebrafish, we identified skeletal abnormalities, including craniofacial cartilage defects and impaired bone development [27]. Therefore, we investigated dysregulated genes associated with the skeletal system that might contribute to these phenotypes.

Several skeletal-related genes were found mostly downregulated in *cdkl5*^−/−^ and many contributed to the significant enrichment of GO terms such as “skeletal system development”, “cartilage development” and “extracellular matrix” both at 5 and 35 dpf. Furthermore, GO terms such as “bone development”, “ossification”, and “regulation of bone mineralization” were only significantly enriched in downregulated genes at 35 dpf. A subset of identified DEGs is listed in Table 4.

Downregulated genes involved in chondrogenesis and cartilage development in *cdkl5*^−/−^ zebrafish included its major regulators *sox6* at both developmental stages and *sox9a* at 5 dpf and *sox9b* at 35dpf, as well as *mbtps1*, *creb3l2*, and *skia* at 5 dpf and *chsy1* at 35 dpf. Expression of the well-known osteogenic markers *sp7* and *runx2a/b* was decreased in *cdkl5*^−/−^ juveniles, as well as genes involved in mineralization such as *tmem119b* at 5 dpf and *tmem119a*, *ano6*, and *ptk2bb* at 35 dpf. Interestingly, genes with both roles in cartilage and bone development were also identified, including *mef2cb* at both developmental stages, *yap1* and *foxl1* at 5 dpf, and *runx1*, *mef2ca*, *prdm1a/b*, *panx3*, *ccn2b*, *pthlha*, *nr3c1* and *rflna/b* at 35 dpf (Table 4).

Dysregulated genes belonging to signaling pathways that are implicated in cartilage development, osteoblast differentiation, and bone formation such as Wntless (Wnt), bone morphogenetic protein (BMP), and transforming growth factor beta (TGFβ) signaling pathways were identified (Table 4). In fact, Wnt-related downregulated genes in *cdkl5*^−/−^ juveniles contributed to the significant enrichment of the Wnt signaling KEGG pathway at 35 dpf. These genes included *wnt7bb*, *wnt7aa*, *wnt16*, *lrp5*, *lrp6*, *dkk2*, *dvl1a*, *dvl2*, *fzd1*, *fzd4*, *frzb*, *ctnnb1*, *ctnnb2*, *bcl9*, *tcf7*, *tle2b/c*, *nkd1*, *nkd2a*, and *kremen1* (Table 4).

A wide diversity of genes encoding components of the ECM crucial for the development and maintenance of cartilage and bone integrity have been identified. Among them, several collagen genes were downregulated in *cdkl5*^−/−^ at both developmental stages, including *col1a1b* and *col1a2*, which are the main constituents of bone ECM, *col5a1*, *col6a2*, *col9a1b*, *col10a1a*, *col11a1b*, and *col12a1a*. The main collagen type in cartilage, *col2a1a*/*b*, was also downregulated at 5 dpf. Other key downregulated ECM constituents included the proteoglycans encoded genes *dcn* at both stages of development, *aspn* at 5 dpf and *hspg2* at 35 dpf; the glycoproteins encoded genes *fn1a*, *fbln2*, and *emilin1b* at 5 dpf and *fbln1* and *comp* at 35 dpf; the thrombospondins encoded genes *thbs1*, *thbs2a*, *thbs3b*, and *thbs4* at both developmental stages. The expression of *spp1* and *bglapl*, two major bone ECM non-collagenous genes, was reduced at 5 and 35 dpf, respectively, in *cdkl5*^−/−^ mutant zebrafish. Furthermore, genes encoding proteins crucial for ECM organization and stabilization through interactions with ECM components were also found to be downregulated, including *hapln1* and *tgfbi* at both developmental stages, *matn1* at 5 dpf, and *vwa1* and *postna/b* at 35 dpf (Table 4).

The expression of genes encoding enzymes of the metalloproteinase family responsible for the degradation of ECM proteins, such as matrix metalloproteinases (MPMs) and a disintegrin and metalloproteinase with thrombospondin motifs (ADAMTS), was reduced in *cdkl5*^−/−^. These included *mmp14a* and *adamts16* at both developmental stages, *mmp15a/b* at 5 dpf and *mmp2*, *mmp9*, *mmp11b*, *mmp13a*, *adamts5*, *adamts7*, *adamts10*, and *adamts15b* at 35dpf. Moreover, at 5 dpf, genes encoding proteases of the cathepsin family were also downregulated in *cdkl5*^−/−^ larvae, including *ctsk*, *ctsl.1*, and *ctsbb*. The expression of the *sh3pxd2b* gene, which encodes an adaptor protein involved in ECM remodeling, also decreased at 5 dpf (Table 4).

Altogether, these results demonstrate that *cdkl5* dysfunction leads to a reduction in genes essential for normal cartilage and bone formation, consistent with the phenotypes observed in *cdkl5*^−/−^ mutant zebrafish.

## 3. Discussion

In humans, pathogenic variants of CDKL5 cause severe developmental and epileptic encephalopathy, accompanied by multisystemic comorbidities as a consequence of CDKL5 deficiency [16]. However, its molecular mechanisms are still poorly understood, particularly those associated with the less studied phenotypes. To decipher potential cdkl5 molecular mechanisms, RNA-seq was performed in the present study to obtain the whole transcriptomic profile of a cdkl5 mutant zebrafish previously identified as a suitable model for CDD [27,55], both at a more initial and at a later stage of development (5 and 35 dpf). The 5 dpf represents a well-established larval stage in zebrafish development, at which most major organ systems, including the nervous system, visual system, and gastrointestinal tract, are functional making it an important time point for assessing early developmental phenotypes and molecularly associated pathways [56,57]. Additionally, at this stage, zebrafish begin exogenous feeding and exhibit a well-characterized locomotor behavior. The 35 dpf corresponds to the juvenile stage, during which zebrafish exhibit more complex behaviors and undergo critical processes related to growth, sexual differentiation, and maturation of organ systems [57]. This stage allows for the evaluation of longer-term effects and the persistence or resolution of phenotypes and altered molecular pathways observed in earlier stages. Together, the 5 dpf and 35 dpf stages offer a temporal framework for analyzing both the earlier and long-term effects of *cdkl5* deficiency. Our findings revealed the dysregulation of several genes in cdkl5^−/−^ mutant zebrafish along with enriched GO functions and pathways implicated in diverse functions and tissues at both stages of development. The most significant DEGs in both conditions were linked to muscle, neuronal, and visual functions, and GO analysis further highlighted the involvement of the DEGs in muscle and neuronal development at both stages and in eye development at 35 dpf. These results indicate a Cdkl5 role within these systems in zebrafish, consistent with the associated phenotypes observed in humans. Although the mechanisms underlying the visual dysfunctions in CDD, such as poor eye contact and lack of visual tracking, are not well understood, they are believed to be linked with cerebral visual impairment [58]. Here, we identified the upregulation of genes crucial for visual perception, including crystalline genes encoding the structural constituent of the eye lens and genes involved in the phototransduction cascade within the retina. Whether this upregulation is due to Cdkl5 loss or a compensatory mechanism in an attempt to counteract potential cerebral visual dysfunctions should be further investigated.

Additionally, GO terms related to ECM organization and actin cytoskeleton regulation were also predominant at both stages. Consistently, KEGG enrichment analysis identified the ECM-receptor interaction and focal adhesion as the most significant downregulated pathways affected by Cdkl5 loss, at both stages of development, and the regulation of the actin cytoskeleton pathway was also enriched at 35 dpf. The ECM, focal adhesions, and actin cytoskeleton form an interconnected system fundamental for tissue development through the regulation of cell adhesion, migration, proliferation, and differentiation, suggesting that their disruption might account for CDD’s underlying mechanism.

### 3.1. Deficiency of Cdkl5 in Zebrafish Causes Alterations in Genes Associated with Muscle System

Individuals with CDD exhibit severely impaired gross motor function with abnormal muscle tone (hypotonia) [16]. Previously, we have also demonstrated that the locomotor behavior of this *cdkl5* mutant zebrafish was impaired, suggesting muscle weakness caused by disturbances in the neurons controlling muscle system functions and/or in the muscle itself. Accordingly, RNA-seq data showed that loss of Cdkl5 led to the downregulation of many key genes involved in muscle development and differentiation that was consistent throughout both developmental stages. Indeed, functional enrichment analysis highlighted the enrichment of muscle development-related GO terms such as muscle cell differentiation and skeletal muscle tissue development. Therefore, our findings suggest a key role of Cdkl5 in normal skeletal muscle development. Nevertheless, Serrano et al. found no obvious defects in the muscle organization or patterning of this *cdkl5* mutant zebrafish at 2 and 6 dpf [55]. Possible reasons for this inconsistency could be the sensitivity of the method used to detect subtle changes or the appearance of defects later in development. To further elucidate the potential structural alterations in the skeletal muscle of *cdkl5* mutant zebrafish, future studies should incorporate additional techniques such as high-resolution immunohistochemistry and electron microscopy.

Interestingly, GO and KEGG pathway analysis revealed the enrichment of downregulated genes associated with cardiac muscle development and cardiac muscle contraction, respectively. To explore potential functional implications, we performed a basic heartbeat assay in embryos at 48 h post-fertilization. This analysis revealed no significant differences in heart rate (beats per minute) between the WT and *cdkl5*^−/−^ mutant zebrafish. However, a more comprehensive characterization of cardiac function in the mutants should be performed. Future studies should employ more robust techniques, such as electrocardiography, which could provide additional information beyond our basic analysis. Furthermore, it is possible that cardiac defects could emerge at later developmental stages not assessed in the current study, and this possibility should be investigated further. Although CDKL5 function in the cardiovascular system is poorly investigated, a few studies have reported cardiac abnormalities in CDD patients and *Cdkl5*^+/−^ female mice, including prolonged QT interval and arrhythmia [59,60].

### 3.2. Cdkl5 Deficiency in Zebrafish Leads to Dysregulation of Genes Involved in Neuronal Functions

Normal brain development requires precise control of neuronal morphogenesis and signaling. Defects in dendrite formation and arborization, axon outgrowth and guidance, and synapse formation result in the dysfunction of neural circuits that are the cause of several neurodevelopmental disorders [61,62,63,64]. A major clinical presentation of CDD is severe neurodevelopmental impairment that affects both cognitive and motor functions. This study revealed that the loss of Cdkl5 in zebrafish led to the dysregulation of several key genes involved in nervous system development at both developmental stages, as well as the contribution of several related GO enrichment functions. Specifically, we observed both the downregulation and upregulation of genes with key roles in neurite outgrowth and axon/dendritic arborization and in axon guidance. We reported the downregulation of genes such as *nfasca*, *zc4h2*, *srgap2*, *prickle1a*, and *prickle2b* which are described as positive regulators of neurite outgrowth and branching, as well as *mycbp2*, which regulates axon extension/guidance and synapse formation [65,66,67,68,69]. Disruption or pathogenic variants in their human orthologs, leading to loss of function, have been associated with neurodevelopmental disorders whose phenotypes resemble CDD, including developmental delay, seizures, motor impairment, hypotonia, intellectual disability, and facial dysmorphism [67,70,71,72,73,74]. Intriguingly, *fez1*, which encodes a protein involved in the regulation of axon and dendrite development [75,76], was found upregulated in *cdkl5*^−/−^ mutant larvae. This finding is unexpected, as previous studies have shown that FEZ1 deficiency leads to neurons with reduced axon length, branching, and dendritic complexity [75,76,77,78]. One possible explanation is that this upregulation represents a compensatory mechanism aimed at counteracting the impaired dendritic and axonal development associated with the loss of Cdkl5. Alternatively, it could suggest dysregulated or altered timing in the neuronal differentiation processes. In addition, Cdkl5 loss in zebrafish caused an alteration in the expression of genes encoding proteins known to interact with or be phosphorylated by CDKL5 in mammals and play a role in these processes. These included IQGAP1 and RAC1 which are crucial regulators of the actin cytoskeleton that together with CDKL5 form a complex involved in dendritic arborization; DLG5 which plays a role in cell adhesion, cell polarity, and transmission of extracellular signals to the cytoskeleton, thereby regulating dendritic spine morphogenesis and synaptogenesis. ARHGEF2, which regulates microtubule and actin cytoskeleton dynamics, as well as focal adhesion, influencing dendritic spine morphology; and SHTN1, which is involved in axon outgrowth [7]. Altogether, our results are consistent with previous studies using other systems such as knockout mice, cultured neurons, and human iPSC-derived neurons, where neuronal CDKL5 loss caused defects in axon outgrowth, dendrite arborization, and synaptogenesis. Therefore, indicating a conservation in CDKL5 molecular mechanisms between mammals and zebrafish.

Since the reduced swimming behavior of *cdkl5*^−/−^ mutant zebrafish might be caused by defects in motor neurons, we have investigated the effect of *cdkl5* dysfunction on the motor neuron development of 3 dpf zebrafish embryos using the *hb9:GFP* transgenic line. At this stage, zebrafish exhibit developed primary and secondary motoneurons that innervate the axial musculature [77,78]. *cdkl5*^−/−^ embryos displayed a reduced number of motor neuron cells and shorter caudal primary axons at 3 dpf, suggesting that loss of Cdkl5 impairs motor neuron development and reduces axonal outgrowth. Accordingly, previous studies showed that CDKL5 knockdown in primary hippocampal neurons and cultured cortical neurons or CDKL5 knockout in hippocampal neurons from mice resulted in a reduction in axons’ total length [8,9,79]. Moreover, Serrano et al. showed that through the crossing with the *islet1:EGFP* line, this *cdkl5* mutant zebrafish presented reduced branching in the middle primary motor neuron at 6 dpf. However, contrary to our findings, Serrano et al. reported no differences in the number of motor neuron cells. This could be due to the distinct markers used to identify motor neurons or the different stages of development that were analyzed. Future studies using whole-mount immunohistochemistry with neuronal-specific markers could be performed to provide better visualization of axon trajectory and detect subtle morphological changes. Altogether, defects in motor neurons, along with the defective expression of genes crucial for muscle development, could explain the impaired locomotor behavior in *cdkl5*^−/−^ mutant zebrafish.

Genes involved in the oligodendrocyte development and myelination were downregulated in *cdkl5*^−/−^ mutant zebrafish from early stages. This glial-cell type is responsible for the formation of the myelin sheath wrapping the axons, that is essential for effective neuronal transmission in the central nervous system [80]. Defects in oligodendrocytes development and maintenance impairing myelination have been implicated with several neurological disorders, including leukodystrophies and autism spectrum disorder (ASD) [81,82,83], that is a feature of CDD. These findings suggest that Cdkl5 might play a role in the differentiation and maturation of oligodendrocytes and myelination in zebrafish. Interestingly, white matter alterations and abnormal myelination were observed in CDD individuals [15]. Nevertheless, the function of CDKL5 in oligodendrocyte development and the myelination process has not yet been investigated.

Precise synaptic transmission is essential for proper brain function. This process begins in the presynaptic neuron, where an action potential opens Ca^2+^ channels. The Ca^2+^ influx triggers neurotransmitters release from the synaptic vesicles (SV) into the synaptic cleft, then interacting with their receptors on the postsynaptic neuron. For this release to happen, the SVs need to be mobilized to the active zone, docked, and primed for membrane fusion [84,85,86]. Abnormalities affecting any of these key modulators of synaptic function have been implicated in the pathogenesis of many neurodevelopmental diseases including epilepsy, intellectual disability, and ASD [87]. Previous studies indicated that *Cdkl5* loss impaired synaptic transmission in mice [88,89,90,91,92,93]. Consistent with this, our transcriptomic analysis revealed that *cdkl5* disruption in zebrafish led to the dysregulation of several genes involved in various steps of the synaptic transmission process, predominantly showing an upregulation pattern. While some of these genes were affected at 5 dpf, most changes occurred later in development, becoming more evident at 35 dpf as the nervous system matures. Genes encoding diverse types of proteins playing crucial roles in the regulation of SV exocytosis and neurotransmission release were overexpressed in *cdkl5*^−/−^ mutant zebrafish. Among these were synaptotagmin genes encoding SV membrane proteins, as well as the vesicle docking regulators *rab3ab* and *rph3ab*, the vesicle priming promoters *rimbp2* and *unc13a*, and the vesicle fusion contributors *snap25a*, *sv2ca*, *stx11b*, and *napb*. Interestingly, a previous study reported an SV2C overexpression in tissue from human epileptic individuals [94]. Moreover, gain-of-function variants in *RPH3A* and *UNC13A* have been detected in individuals with phenotypes ranging from epilepsy and developmental delay to ASD [95,96]. These phenotypes are part of the main clinical presentation of individuals with CDKL5 deficiency disorder and an increased susceptibility to seizures was observed in this *cdkl5*^−/−^ zebrafish mutant. Therefore, our results suggest that Cdkl5 plays a role in presynaptic function and its deficiency in zebrafish might enhance SV exocytosis, resulting in aberrant neurotransmitter release thus impairing neuronal transmission. So far, few studies have investigated the presynaptic role of CDKL5, and its only known presynaptic target is amphiphysin 1 that is involved in SV endocytosis and recycling [9]. Additionally, it was reported that loss of CDKL5 in primary hippocampal neurons of rats led to slower SV endocytosis, while SV exocytosis remained unaffected [97]. This inconsistency may be due to differences in the specific cell type analyzed, the developmental stage studied, or even species differences.

Ion channels are also known to play a crucial role in synaptic neurotransmission. Their abnormal activity, whether caused by gain- or loss-of-function (GOF; LOF), is associated with numerous diseases classified as channelopathies [98,99]. Here, we identified several DEGs encoding both voltage- and ligand-gated ion channels, most of which were overexpressed in *cdkl5*^−/−^ mutant zebrafish. Voltage-gated calcium channels control the Ca^2+^ influx into cells triggered by membrane depolarization. We observed the upregulation of genes such as *cacna1aa*, *cacna1c*, *cacna1da* encoding the calcium channel subunits Cav2.1, Cav1.2, and Cav1.3, whose GOF mutations in humans lead to excessive Ca^2+^ entry and increased neuron excitability causing developmental disorders such as epilepsy or ASD [100,101,102]. Recently, the Cav2.3 subunit encoded by *CACNA1E* was identified as a target of CDKL5 in humans and mice, and loss of its phosphorylation leads to channel GOF causing increased calcium influx and increased neuronal excitability [103]. Additionally, we identified the upregulation of voltage-gated sodium channel genes with key roles in the generation and propagation of action potentials, including *scn8aa/b*, *scn1ba/b* genes whose mutations in humans are associated with developmental and epileptic encephalopathies (DEE) [104,105,106,107]. Typically, mutations in *SCN8A* result in the gain of function, causing higher sodium currents and channel hyperactivity [105,106,108]. Interestingly, a recent study showed the effectiveness of sodium channel blockers to control seizures in CDD individuals [109]. Furthermore, we have also observed the upregulation of voltage-gated sodium channel genes such as *kcna1a*, *kcnq3*, *kcnb1*, *kcnc1b*, and *kcnd1* whose orthologs in humans have been implicated in epilepsy syndromes like DEE by either LOF or GOF mutations [110,111,112,113,114,115]. Accordingly, organoids derived from CDD individuals displayed neuronal hyperexcitability due to voltage-gated channel dysfunction, marked by increased Na^+^ and K^+^ current densities and premature Na^+^ channel opening [116]. This might suggest common molecular mechanisms for the seizure pathophysiology in CDD between fish and mammals. This study also revealed the overexpression of ligand-gated ion channels in *cdkl5*^−/−^ zebrafish comprising both glutamate and GABA neurotransmitter receptors, that mediate excitatory (E) and inhibitory (I) synaptic neurotransmission, respectively. The impaired function of these types of receptors leading to disrupted E/I balance is linked with neurodevelopmental disorders [117]. Among the identified upregulated genes were the glutamate receptors GRIN1A and *gria3a/b* and the GABA receptor *gabra1*, *gabrd*, and *gabrg2* whose both LOF and GOF mutations in human orthologs are known to cause neurodevelopmental phenotypes including epilepsy, cognitive impairment and/or ASD [118,119,120,121,122,123,124]. This study suggests that loss of Cdkl5 in zebrafish enhances glutamatergic and GABAergic synaptic neurotransmission. In accordance, research using CDKL5 mouse models has revealed disturbances in the E/I balance across multiple brain regions. Consistently, overexpression of glutamate receptors has been reported, leading to enhanced glutamatergic neurotransmission [91,92,125]. Selective loss of CDKL5 in forebrain glutamatergic neurons triggered spontaneous seizures in conditional Nex-cKO mice, due to increased excitability in dentate gyrus granule cells (DGCs), accompanied by increased inhibitory activity, which may be a compensatory response to counteract the E/I imbalance [89]. Similar observations were made in CA1 pyramidal neurons, following CDKL5 ablation in forebrain glutamatergic neurons of *Cdkl5* Emx1-cKO mice, leading to hippocampal-associated learning and memory deficits [93]. In contrast, CDKL5 deficiency in forebrain GABAergic neurons resulted in enhanced glutamatergic transmission and hypercitability in CA1 pyramidal neurons, leading to autistic features [92]. In addition, CDKL5 loss reduced and augmented excitatory and inhibitory transmission, respectively, decreasing the E/I ration in DGCs of *Cdkl5*^−/y^ mice thus impairing hippocampal learning and memory [90]. Increased inhibitory GABAergic transmission was also reported in the primary visual cortex and perirhinal cortex of *Cdkl5*^−/y^ knockout mice, which might underline the CDD visual impairments and novel object recognition memory deficits, respectively [88,126]. Altogether, our findings indicate a CDKL5 role in the regulation of ion channels’ activity, supporting prior studies stating that CDD is in part a channelopathy.

### 3.3. Loss of Cdkl5 in Zebrafish Disrupts the Expression of Genes Associated with the Skeletal System

Discrete skeletal phenotypes have been observed in CDD individuals, such as dysmorphic facial features and scoliosis [16,18]. Nevertheless, the mechanisms underlying their occurrence are still not known, as no investigations have been conducted to specifically address this issue. In our previous work, we identified skeletal abnormalities in *cdkl5*^−/−^ mutant zebrafish using alcian blue and alizarin red staining. These included shortened craniofacial cartilage structures and decreased bone mineralization [27]. In the present study, we gained insights into the molecular mechanisms underlying these defects.

During development, cartilage is the first skeletal tissue to form, a process that occurs when mesenchymal stem cells (MSCs) differentiate into chondrocytes, which produce an ECM [127,128,129,130]. Bone formation initiates with MSCs and can occur through different forms involving osteoblast deposition of an ECM and its later mineralization [128,129,130]. At 5 dpf, the major craniofacial cartilages, including Meckel’s cartilage, palatoquadrate, and ceratohyal, are formed, while the bone formation starts [130,131]. At 35 dpf, the bone structure of the axial skeleton is fully developed [132]. In line with the previously observed impaired cartilage and bone formation in *cdkl5*^−/−^ mutants, GO enrichment analysis revealed enrichment of downregulated DEGs involved in cartilage development from the earliest developmental stage analyzed, as well as in bone development, ossification, and mineralization at the later stage. Loss of Cdkl5 reduced the expression of master regulators of early chondrogenesis like *sox9* and *sox6*, which are crucial for chondrocyte proliferation and differentiation by inducing the expression of cartilaginous ECM components [127]. Consistently, the expression of main cartilage ECM components was impaired including the collagen genes *col2a1*, *col9a1b*, and *col11a1* whose expression is known to be regulated by SOX9 [133,134,135], as well as proteoglycans and glycoproteins encoding genes. Abnormal ECM synthesis and composition have been linked to several diseases associated with craniofacial malformation and skeletal defects [136,137]. Overall, the altered craniofacial cartilage structures in the *cdkl5*^−/−^ mutant zebrafish might be the result of a decreased expression of crucial regulators of chondrogenesis and cartilage ECM constituents supporting our previous hypothesis that Cdkl5 plays an important role in cartilage formation and development. Moreover, we observed reduced expression of *col10a1* and genes encoding ECM-degrading proteases such as *mmp13*, *mmp9*, and *adamts5* which are well-established markers of terminally differentiated hypertrophic chondrocytes [138,139,140]. This suggests that Cdkl5 loss impairs endochondral ossification, as the expression of these genes in hypertrophic chondrocytes is required for cartilage replacement by bone.

Intramembranous ossification, which occurs through the direct differentiation of MSCs into mature osteoblasts capable of producing bone ECM [130], also appears to be regulated by Cdkl5 in zebrafish. Loss of Cdkl5 resulted in decreased expression of key osteogenic markers, including *runx2* and *sp7*, which encode transcription factors crucial for early differentiation from MSCs to pre-osteoblasts, as well as major bone ECM-encoding genes such as the early osteoblast marker *col1a1b*, the middle-to-late marker *spp1* and the late marker *bglapl* [141]. Signaling pathways involved in skeletal development were affected in *cdkl5*^−/−^ mutant zebrafish, particularly the Wnt signaling pathway, which was significantly downregulated at 35 dpf, as determined by KEGG analysis. Studies in human and animal models have demonstrated that Wnt signaling induces bone formation, and its dysregulation is associated with bone disorders such as osteoporosis [142,143,144,145]. These findings indicate that the impaired or delayed mineralization phenotype of *cdkl5*^−/−^ mutant zebrafish results from defective osteogenic differentiation, suggesting that Cdkl5 plays an important role in bone development.

## 4. Materials and Methods

### 4.1. Ethics Statement

All animal procedures carried out in the present study were performed in compliance with ARRIVE guidelines (https://arriveguidelines.org (accessed on 15 January 2021)) and according to the EU and Portuguese legislation for animal experimentation and welfare (Directives 86/609 CEE and 2010/63/EU; Decreto-Lei 113/2013; Portaria1005/92, 466/95 and 1131/97). This study was approved by the Portuguese Direção-Geral de Alimentação e Veterinária (authorization no. 0421/2021). Qualified operators conducted animal handling and experimentation, prioritizing the minimization of pain, distress, and discomfort.

### 4.2. Fish Maintenance

Experiments were conducted using the *cdkl5*^sa21938^ mutant zebrafish line (obtained through the European Zebrafish Resource Center) and their wild-type (WT) siblings. All adult zebrafish and larvae were maintained in the animal facility in a recirculating water system under controlled temperature (28 °C) and lighting (14h light 10 h dark cycle). For the experiments, homozygous *cdkl5*^sa21938^ (*cdkl5*^−/−^) mutant adult fish were incrossed to generate *cdkl5*^−/−^ embryos, while WT embryos were obtained by crossing adult WT siblings, which were the offspring of heterozygous *cdkl5*^sa21938^ mutant incrosses. The *cdkl5*^−/−^ fish were also crossed with Tg(Hb9:GFP) transgenic reporter fish line [146,147]. The resulting heterozygous zebrafish were incrossed to obtain the homozygous lines, which were confirmed by genotyping. Zebrafish were allocated to experimental groups based on their genotype. Animal care technicians were unaware of the allocation groups to ensure that all animals in the experiment were handled, monitored, and treated in the same way.

### 4.3. RNA Extraction

Total RNA was isolated from larvae and juveniles of homozygous cdkl5*^sa21938^* and their WT siblings at 5 and 35 days post-fertilization, respectively, using the NZYOL reagent (NZYTech, Lisbon, Portugal) and following the manufacturer’s instructions. Each group comprised five biological replicates, prepared from pools of 50 larvae or 7 juveniles. The number of replicates was selected to ensure adequate statistical power, while the number of embryos per replicate was determined based on the RNA quantity required for RNA-seq and RT-qPCR analyses. Embryos and larvae of different replicates originated from different parents. DNase treatment was then performed using the RNase-Free DNase set (QIAGEN, Hilden, Germany) to eliminate any remaining genomic DNA contamination. To increase purity, RNA samples were subjected to the RNeasy kit (QIAGEN) clean-up protocol. The RNA quantity and purity were assessed using a Nanodrop spectrophotometer (Thermo Fisher Scientific, Waltham, MA, USA), and its integrity was verified by electrophoresis on a 1% agarose gel.

### 4.4. RNA Sequencing (RNA-Seq)

The purified RNA was delivered to Novogene company (Cambridge, UK) for library construction and sequencing. Integrity of RNA samples was analyzed on the Agilent 2100 bioanalyzer (Santa Clara, CA, USA) and only samples with an RNA integrity number (RIN) > 8 were further used for cDNA library preparation. Briefly, mRNA was separated from total RNA using poly-T oligo-attached magnetic beads and subjected to fragmentation using divalent cations under high temperatures. Then, the first strand cDNA was synthesized using random hexamer primers and M-MuLV Reverse Transcriptase, followed by the second strand cDNA synthesis using DNA polymerase I and RNase H. The lasting overhangs were transformed into blunt ends through polymerase and exonuclease reactions. To prepare for hybridization, adaptors containing a hairpin loop structure were ligated to the previously 3′ ends adenylated DNA fragments. The AMPure XP system (Beckman Coulter, Beverly, CA, USA) was used to purify the cDNA fragments with 370~420 bp in length that were then digested with USER enzyme. PCR was carried out using Phusion High-Fidelity DNA polymerase, universal PCR primers and Index (X) primer. The PCR products were purified, and the Agilent Bioanalyzer 2100 system was used to evaluate the library quality. Finally, directional (strand-specific) libraries were sequenced on the Illumina Novaseq platform, generating 150-bp paired-end reads for each sample.

### 4.5. Bioinformatic Analysis of RNA-Seq Data

For the RNA-Seq data analysis, raw reads in fastq format were first trimmed to obtain clean reads through the elimination of low-quality reads, adapter-containing reads, and ploy-N-containing reads. After filtering, clean reads were aligned to the zebrafish reference genome (*Danio rerio* GRCz11) using Hisat2 (v2.0.5). For gene expression quantification, featureCounts (v1.5.0-p3) was used to count the number of reads mapped to each gene. Fragments per kilobase per million mapped fragments (FPKM) for each gene were then calculated, considering the gene length and the total number of mapped reads. Genes with FPKM values greater than 1 were considered to be expressed. Analysis of the differential expression between *cdkl5* mutant and WT zebrafish was performed using the DESeq2 R package (1.20.0). The resulting *p*-values were adjusted (padj) according to the method proposed by Benjamini and Hochberg [148] for controlling the false discovery rate. Genes with a *p*-value < 0.05 and |log2(FoldChange)| > 0 found by DESeq2 were considered as differentially expressed. Gene Ontology (GO) enrichment analysis of differentially expressed genes (DEGs) was performed using the clusterProfiler R package. GO terms with corrected *p*-value < 0.05 were considered significantly enriched by differentially expressed genes. Kyoto Encyclopedia of Genes and Genomes (KEGG) analysis was performed to identify significantly enriched pathways associated with DEGs. The clusterProfiler package in R was used to test the statistical enrichment of DEGs in KEGG pathways.

### 4.6. cDNA Synthesis

Total RNA was reverse-transcribed into cDNA using the Moloney murine leukemia virus (M-MLV) reverse transcriptase (Invitrogen, Carlsbad, CA, USA). Briefly, 1 µg of RNA was combined with 0.4 µL of oligo(dT) primer (50 mM), 1 µL of dNTPs (10 mM), and DNase/RNase-free water to a final volume of 12 µL, then incubated at 65 °C for 5 min. Next, 4 µL of 5× first strand buffer, 2 µL of DTT, and 1 µL of Ribolock RNase inhibitor were added, followed by incubation at 37 °C for 2 min. Subsequently, 1 µL of M-MLV was added, and the reverse transcription continued for 50 min at 37 °C. Finally, the mixture was incubated at 70 °C for 15 min to inactivate the enzyme.

### 4.7. Quantitative Real-Time PCR (qPCR)

Gene-specific amplifications by qPCR were performed in 20 µL reactions containing 10 µL of SensiFAST SYBR Hi-ROX (Meridian Bioscience, Cincinnati, OH, USA), 0.8 µL of each forward and reverse set of primers (10 µM) and 2 μL of cDNA (diluted 1:10). Gene-specific primers are listed in Supplementary Table S12. The qPCR reactions were carried out on a 96-well plate, using a CFX Connect Real-Time PCR Thermocycler (Biorad, Hercules, CA, USA) and under the following conditions: an initial denaturation step at 95 °C for 2 min, followed by 40 cycles of amplification (each cycle is 5 s at 95 °C, 20 s at 60 °C). After the amplification was completed, a melting curve was generated by gradually heating the sample to 95 °C at a rate of 0.1 °C per second, while continuously capturing the fluorescence. For each sample, at least two technical replicates were performed. Relative gene expression levels were determined by applying the ΔΔCt comparative method and were normalized to the β-actin and *rps18* reference genes for 5 dpf and 35 dpf samples, respectively (Supplementary Table S12).

### 4.8. Imaging and Analysis of the Tg(hb9: GFP) Embryos

Embryos with 3 dpf were anesthetized in MS-222, oriented on the lateral side on top of 0.8% agarose, and fluorescent images were acquired using the ZEISS AXIO Zoom V16 microscope. Images were captured and processed using ZEN 3.7 software. The deconvolution function (deblurring) was used to improve resolution, using the same parameters in all images. The number of GFP^+^ spinal motor neurons present in three hemisegments after the distal end of the yolk extension was quantified. The length of five motor axons (located immediately after the yolk sac, over the yolk extension) per embryo was measured using the Neuroanatomy/SNT plug-in [149] for ImageJ version 1.54p/Fiji version 2.16.0. The average of the five values was calculated for subsequent statistical analysis.

### 4.9. Statistical Analysis

Statistical analysis was performed using Prism version 8 (GraphPad Software). The Kolmogorov-Smirnov test was used to determine the normality of the data. Data with a normal distribution are presented as mean ± SD. *t*-test with Welch’s correction was used to identify significant differences between two groups. Differences were considered statistically significant when *p* < 0.05. Blind analyses were performed to avoid the bias associated with experimenters.

## 5. Conclusions

In summary, this study contributes to gaining insights into the Cdkl5 molecular mechanisms through the identification of genes, functions, and pathways affected by its disruption. Overall, our findings highlight the key role of Cdkl5 in neuronal development and synaptic neurotransmission, suggesting a common Cdkl5 function between zebrafish and mammalian models. Moreover, we demonstrate that *cdkl5* disruption causes motor neuron defects that in combination with abnormal muscle development and structure may contribute to the observed gross motor impairments. Finally, this study provides novel evidence of Cdkl5’s role in cartilage and bone development, potentially underlying the skeletal defects observed in mutant zebrafish and individuals with CDD. Altogether, these results emphasize the *cdkl5*^−/−^ mutant zebrafish as a valuable model for CDD and highlight its potential for screening novel drugs to treat the disorder’s symptoms.

## Figures and Tables

**Table 1:** Summary of RNA-seq data obtained from each analyzed sample of *cdkl5*^−/−^ and WT sibling (WT) zebrafish at 5 and 35 days post-fertilization.

Samples ^a^	Raw Reads ^b^	Clean Reads ^c^	Error Rate (%) ^d^	Q30 (%) ^e^	GC Content (%) ^f^	Total Mapped Reads ^g^	Mapping Rate (%) ^h^
5-day post-fertilization larvae							
WT_1	44,243,738	43,112,532	0.3	92.57	47.16	39,424,978	91.45
WT_2	42,756,808	42,556,832	0.3	92.77	46.37	38,839,634	91.27
WT_3	40,611,946	39,916,404	0.3	93.45	47.45	36,887,037	92.41
WT_4	42,441,392	41,953,914	0.3	93.11	47.13	38,545,430	91.88
WT_5	46,901,404	46,660,422	0.3	93.29	46.42	42,640,932	91.39
*cdkl5*^−/−^_1	40,735,320	40,073,286	0.3	93.52	46.84	36,410,981	90.86
*cdkl5*^−/−^_2	52,177,484	51,304,154	0.3	93.30	46.93	47,301,907	92.20
*cdkl5*^−/−^_3	60,766,130	59,824,088	0.3	93.25	47.10	55,136,748	92.16
*cdkl5*^−/−^_4	51,224,048	50,335,178	0.3	93.33	47.02	46,424,268	92.23
*cdkl5*^−/−^_5	44,166,960	43,928,492	0.3	93.76	46.67	40,510,839	92.22
35 days post-fertilization juveniles							
WT_1	47,686,434	46,651,198	0.01	94.81	47.77	41,569,695	89.11
WT_2	40,746,140	39,968.148	0.01	95.19	47.68	35,485,612	88.78
WT_3	50,558,002	49,544,238	0.01	94.95	47.06	43,648,555	88.1
WT_4	42,848,716	41,808,602	0.01	94.18	47.08	36,540,299	87.4
WT_5	45,307,966	44,264,802	0.01	93.72	47.30	39,159,487	88.47
*cdkl5*^−/−^_1	42,268,238	41,557,848	0.01	93.72	47.30	36.707,847	88.33
*cdkl5*^−/−^_2	47,642,904	46,602,930	0.01	94.69	47.35	41,246,902	88.51
*cdkl5*^−/−^_3	41,748,936	40,889,454	0.01	93.88	47.21	36,054,652	88.18
*cdkl5*^−/−^_4	49,092,294	47,927,570	0.01	94.83	47.14	42,443,230	88.56
*cdkl5*^−/−^_5	50,491,206	49,358,790	0.01	94.81	47.27	43,353,446	87.83

^a^ 1, 2, 3, 4, and 5 indicate the five biological replicates. ^b^ Raw reads: total number of reads obtained before processing. ^c^ Clean reads: number of reads after filtering of raw reads. ^d^ Error rate: base error rate of whole sequencing. ^e^ Q30: percentage of bases with quality score greater than 30, i.e., with accuracy over 99.9%. ^f^ GC content: percentage of G and C bases in total number of bases. ^g^ Total mapped reads: the number of clean reads that mapped onto the reference genome. ^h^ Mapping rate: percentage of clean reads aligned to the reference genome.

**Table 2:** The top 20 known upregulated and downregulated differentially expressed genes between *cdkl5*^−/−^ mutant and WT siblings’ zebrafish at 5 and 35 days post-fertilization. For each gene is reported the log2(fold change) of *cdkl5*^−/−^ over WT, the *p*-value and corrected *p*-value (padj) calculated by DESeq2.

Gene Symbol	Protein Name	Log2(Fold Change)	*p*-Value	Padj
5 days post-fertilization larvae				
*sort1a*	Sortilin 1a	1.8794	2.9996 × 10^−13^	1.1657 × 10^−9^
*tdo2b*	Tryptophan 2,3-Dioxygenase 2b	0.8033	9.0703 × 10^−11^	1.9583 × 10^−7^
*cflara*	CASP8 and FADD-like apoptosis regulator a	0.7984	1.3040 × 10^−10^	2.5339 × 10^−7^
*ube2l3a*	Ubiquitin-conjugating enzyme E2L 3a	2.2685	4.5907 × 10^−10^	6.8616 × 10^−7^
*pdap1a*	pdgfa associated protein 1a	0.8095	1.3519 × 10^−8^	1.3135 × 10^−5^
*csrp2*	Cysteine and glycine-rich protein 2	0.7129	1.4895 × 10^−8^	1.3782 × 10^−5^
*igfbp1b*	Insulin-like growth factor binding protein 1b	0.9922	2.3254 × 10^−8^	2.0442 × 10^−5^
*guca1c*	Guanylate cyclase activator 1C	0.9678	2.9697 × 10^−8^	2.4044 × 10^−5^
*nts*	Neurotensin	4.3662	1.9960 × 10^−7^	0.0001
*tmem240b*	Transmembrane protein 240b	1.1948	2.8351 × 10^−7^	0.0002
*ndufs7*	NADH Dehydrogenase [Ubiquinone] Iron-Sulfur Protein 7	1.0968	3.1092 × 10^−7^	0.0002
*c7b*	Complement component 7b	1.7532	3.7864 × 10^−7^	0.0002
*mt2*	Metallothionein 2	1.2515	9.1100 × 10^−7^	0.0005
*phf13*	PHD finger protein 13	0.5807	2.5537 × 10^−6^	0.0012
*krt15*	Keratin 15	0.9521	2.9615 × 10^−6^	0.0013
*cspg4bb*	chondroitin sulfate proteoglycan 4bb	1.7450	4.0039 × 10^−6^	0.0017
*ccdc149a*	coiled-coil domain containing 149a	2.0997	5.2690 × 10^−6^	0.0021
*arr3b*	Arrestin 3b	0.7502	5.3098 × 10^−6^	0.0021
*tcnba*	Transcobalamin beta a	0.7376	7.0446 × 10^−6^	0.0024
*cel.2*	Carboxyl ester lipase, tandem duplicate 2	0.5815	8.2700 × 10^−6^	0.0028
*gamt*	Guanidinoacetate N-methyltransferase	−3.1079	1.1615 × 10^−92^	2.2569 × 10^−88^
*nucks1a*	Nuclear casein kinase and cyclin-dependent kinase substrate 1a	−1.3425	5.4136 × 10^−19^	5.2596 × 10^−15^
*pir*	Pirin	−1.3499	3.3308 × 10^−17^	2.1574 × 10^−13^
*ctsl.1*	Cathepsin L.1	−1.2024	2.3532 × 10^−13^	1.1431 × 10^−9^
*card19*	Caspase recruitment domain family member 19	−1.5736	8.6568 × 10^−12^	2.8035 × 10^−8^
*ugt1b5*	UDP glucuronosyltransferase 1 family, polypeptide B5	−1.8045	2.4384 × 10^−11^	6.7686 × 10^−8^
*dynlt3*	Dynein Light Chain Tctex-Type 3	−0.8301	1.6882 × 10^−10^	2.9821 × 10^−7^
*klhdc8a*	Kelch domain containing 8A	−6.1579	2.4233 × 10^−10^	3.9239 × 10^−7^
*ccdc120*	Coiled-coil domain containing 120	−1.9919	6.3624 × 10^−10^	8.8305 × 10^−7^
*foxj3*	Forkhead box J3	−0.7362	9.5553 × 10^−10^	1.2378 × 10^−6^
*slc15a2*	Solute carrier family 15, member 2	−0.8038	8.1421 × 10^−9^	8.3268 × 10^−6^
*slc12a10.1*	Solute carrier family 12, member 10, tandem duplicate 1	−1.8439	2.4197 × 10^−8^	2.0442 × 10^−5^
*cishb*	Cytokine inducible SH2-containing protein b	−1.8439	7.1115 × 10^−8^	5.4873 × 10^−5^
*mbd1b*	Methyl-CpG binding domain protein 1b	−1.1586	7.3423 × 10^−8^	5.4873 × 10^−5^
*slc41a1*	Solute carrier family 41, member 1	−4.4974	2.1192 × 10^−7^	0.0001
*rs1a*	Retinoschisin 1a	−0.5785	2.2400 × 10^−7^	0.0001
*etnk2*	Ethanolamine kinase 2	−5.2767	3.2897 × 10^−7^	0.0002
*lrp2b*	Low-density lipoprotein receptor-related protein 2b	−1.2138	5.5833 × 10^−7^	0.0003
*gnl3l*	G protein nucleolar 3 like	−2.7313	8.3341 × 10^−7^	0.0004
*myh7ba*	Myosin heavy chain 7B	−0.8244	1.2271 × 10^−6^	0.0006
*col5a1*	Collagen type V alpha 1 chain	−0.4288	1.2532 × 10^−6^	0.0006
35 days post-fertilization juveniles				
*olfml3b*	Olfactomedin-like 3b	5.2512	1.5620 × 10^−78^	1.6759 × 10^−74^
*kdm5ba*	Lysine Demethylase 5Ba	1.5014	3.4759 × 10^−16^	6.2161 × 10^−13^
*crygm2d18*	crystallin, gamma M2d18	2.4337	7.8911 × 10^−15^	1.2096 × 10^−11^
*ipo9*	Importin 9	2.0313	8.7711 × 10^−15^	1.2549 × 10^−11^
*rlbp1b*	Retinaldehyde binding protein 1b	1.9842	2.3868 × 10^−14^	3.2013 × 10^−11^
*ggctb*	Gamma-glutamylcyclotransferase b	1.0173	3.8752 × 10^−12^	4.3769 × 10^−9^
*lim2.4*	Lens intrinsic membrane protein 2.4	1.2252	1.4216 × 10^−11^	1.4528 × 10^−8^
*tfcp2l1*	Transcription factor CP2-like 1	1.1479	4.2758 × 10^−11^	3.6703 × 10^−8^
*krt15*	Keratin 15	1.5442	1.1659 × 10^−10^	9.0605 × 10^−8^
*crygm2d15*	Crystallin gamma M2d15	2.2803	1.2171 × 10^−10^	9.0605 × 10^−8^
*crygm2d16*	Crystallin gamma M2d16	2.3116	1.2224 × 10^−10^	9.0605 × 10^−8^
*crygm2d11*	Crystallin gamma M2d11	1.5472	1.2244 × 10^−10^	9.0605 × 10^−8^
*rrp9*	Ribosomal RNA processing 9	1.6369	1.4287 × 10^−10^	9.8906 × 10^−8^
*cryba2a*	Crystallin beta a2a	1.1551	2.3574 × 10^−10^	1.5809 × 10^−7^
*crygm2d13*	Crystallin gamma M2d13	1.6363	4.4261 × 10^−10^	2.7139 × 10^−7^
*parapinopsinb*	Parapinopsin b	2.9711	6.3436 × 10^−10^	3.5825 × 10^−7^
*foxq1a*	Forkhead box Q1a	0.6750	7.2768 × 10^−10^	3.9040 × 10^−7^
*dynlrb1*	Dynein light chain roadblock-type 1	0.8483	8.6657 × 10^−10^	4.5358 × 10^−7^
*timm17a*	Translocase of inner mitochondrial membrane 17A	1.4058	2.2971 × 10^−9^	1.0060 × 10^−6^
*crygm2d14*	Crystallin, gamma M2d14	1.8210	2.5513 × 10^−9^	1.0950 × 10^−6^
*opn1lw2*	Opsin 1, long-wave-sensitive, 2	−4.8460	4.6970 × 10^−108^	1.0080 × 10^−103^
*opn1lw1*	Opsin 1, long-wave-sensitive, 1	−4.5892	3.4828 × 10^−70^	2.4914 × 10^−66^
*gnl3l*	G protein nucleolar 3 like	−3.1493	4.3148 × 10^−58^	2.3149 × 10^−54^
*nucks1a*	Nuclear casein kinase and cyclin-dependent kinase substrate 1a	−1.4428	4.5028 × 10^−30^	1.7153 × 10^−26^
*pir*	Pirin	−1.9600	9.5388 × 10^−29^	2.9243 × 10^−25^
*slc41a1*	Solute carrier family 41, member 1	−4.2114	2.8678 × 10^−26^	7.6930 × 10^−23^
*ccdc120*	Coiled-coil domain containing 120	−2.7935	6.8052 × 10^−23^	1.6227 × 10^−19^
*ugt1b5*	UDP glucuronosyltransferase 1 family, polypeptide B5	−1.4197	1.1478 × 10^−18^	2.4631 × 10^−15^
*dynlt3*	Dynein Light Chain Tctex-Type 3	−0.8398	1.6501 × 10^−17^	3.2191 × 10^−14^
*nfasca*	Neurofascin a	−1.0500	1.0572 × 10^−12^	1.3345 × 10^−9^
*mbd1b*	Methyl-CpG binding domain protein 1b	−1.6633	3.8504 × 10^−12^	4.3769 × 10^−9^
*myh7ba*	Myosin heavy chain 7B	−0.8647	3.0928 × 10^−11^	2.8019 × 10^−8^
*ehd2b*	EH-domain containing 2b	−0.4425	1.2915 × 10^−10^	9.2386 × 10^−8^
*igfn1.1*	immunoglobulin-like and fibronectin type III domain containing 1, tandem duplicate 1	−1.1561	4.2629 × 10^−10^	2.7139 × 10^−7^
*cishb*	Cytokine inducible SH2-containing protein b	−1.6556	4.3495 × 10^−10^	2.7139 × 10^−7^
*htra1b*	HtrA serine peptidase 1b	−0.6314	4.9847 × 10^−10^	2.8959 × 10^−7^
*myhc4*	Myosin heavy chain 4	−1.5783	6.6454 × 10^−10^	3.6570 × 10^−7^
*vgll2b*	Vestigial-like family member 2b	−1.2804	1.3015 × 10^−9^	6.3616 × 10^−7^
*myom2a*	Myomesin 2a	−1.1321	1.3043 × 10^−9^	6.3616 × 10^−7^
*vwa1*	von Willebrand factor A domain containing 1	−0.5727	1.4088 × 10^−9^	6.7183 × 10^−7^

**Table 3:** Subset of differentially expressed genes related to neuronal functions in *cdkl5*^−/−^ vs. WT zebrafish at 5 dpf, 35 dpf, and both developmental stages.

Functions	Genes Name		
	5 dpf	35 dpf	Both Stages
Neuronal morphogenesis, neurite outgrowth, dendritic arborization, axon guidance	**Downregulated**: *slit1a*, *cntf*, *casp3a*, *pcdh18b*, *mycbp2, zc4h2*, *arhgef2*, *dlg5a*, *rac1b* **Upregulated**: *draxin*, *nefmb*, *nefla*, *stmn3, stmn4*, *srcin1b, amigo1*, *ndr2*, *fez1*, *shtn1*	**Downregulated**: *slit2*, *pax6b*, *srgap2*, *prickle1a*, *prickle2b*, *smad3a*	**Downregulated**: *nfasca*, *ntn1a*, *nav2b*, *plxnb2a*, *iqgap1* **Upregulated**: *stmn1b*, *pacsin1a*
Oligodendrocyte development and myelination	**Upregulated**: *plp1b*	**Downregulated**: *plp1b*, *lama2, actr10*	**Downregulated**: *tfeb*, *notch3*, *prx*, *daam2*
Synaptic signaling, neurotransmitters secretion/transport, ion channels	**Downregulated**: *cacna1hb*, *cacng6b* **Upregulated**: *cacng5a, cacng3b*, *cacng7b*, *kcnb1*, *gabrr3a*, *gabrb1*, *grm2b*, *grm5a*	**Downregulated**: *sntb1*, *snap47*, *syt6a*, *cacna1g*, *cacnb1*, *grm6b*, *grinab* **Upregulated**: *syt2a*, *syt6b, syt11b*, *rab3ab*, *rph3ab*, *rimbp2*, *unc13a*, *sv2ca*, *napbb*, *lrrc4bb*, *syngap1a*, *drd2b*, *cacna1aa*, *cacna1da*, *cacnb4a*, *cacna1c*, *cacna2d3*, *scn8aa/b*, *scn1lab*, *nalcn, scn1ba*, *kcna1a*, *kcnq3*, *kcnc1b gabrg1*, *gabrb4*, *gabra1*, *gabrg2*, *gabbr1a*, *grin1a*, *gria3a/b*, *grin3a*, *glrba*	**Downregulated**: *rapsn*, *cacna2d1a, scn4ab*, *gabra6b*, *chrna1*, *chrne* **Upregulated**: *syt12*, *snap25a*, *stx12l*, *scn1bb*, *kcnj3b, kcnd1*, *gabrd*

**Table 4:** Subset of differentially expressed genes related to cartilage and bone functions found in *cdkl5*^−/−^ vs. WT zebrafish at 5 dpf, 35 dpf, and both developmental stages.

Functions/Pathways	Genes Name		
	5 dpf	35 dpf	Both Stages
Chondrogenesis	**Downregulated**: *sox9a*, *mbtps1*, *creb3l2*, *skia*	**Downregulated**: *sox9b, chsy1*	**Downregulated**: *sox6*
Osteogenesis	**Downregulated**: *tmem119b*	**Downregulated**: *sp7*, *runx2a/b*, *tmem119a, ano6*, *ptk2bb*	
Cartilage and Bone development	**Downregulated**: *yap1*, *foxl1*	**Downregulated**: *runx1*, *mef2ca*, *prdm1a/b*, *panx3*, *ccn2b*, *pthlha*, *nr3c1, rflna/b*	**Downregulated**: *mef2cb*
Wntless (Wnt), bone morphogenetic protein (BMP), and transforming growth factor beta (TGFβ) signaling pathways	**Downregulated**: *wisp1b*, wls, *sfrp1a*, *notum1a*, *bmper* **Upregulated**: *wnt7ba*, *wnt6a*, *dkk1b, bmp2b*,	**Downregulated**: *wnt7bb*, *wnt7aa*, *wnt16*, *lrp5*, *lrp6*, *dkk2*, *dvl1a*, *fzd1*, *frzb*, *ctnnb1*, *ctnnb2*, *bcl9*, *tcf7*, *tle2b/c*, *nkd1*, *nkd2a*, *bmp1a*, *bmp8a*, *tgfb3*, *tgfbr3, tgfbr2a*	**Downregulated**: *dvl2*, *fzd4*, *kremen1*
Extracellular matrix (ECM) constituents and organization	**Downregulated**: *col2a1a/b*, *aspn*, *fn1a*, *fbln2*, *emilin1b*, *spp1*, *matn1*, *thbs1b*, *thbs4a*, *hapln1a*	**Downregulated**: *hspg2*, *comp*, *postna/b*, *fbln1*, *thbs1a*, *thbs4b*, *bglapl*, *hapln1b*, *vwa1*	**Downregulated**: *col1a1b*, *col1a2*, *col5a1*, *col6a2*, *col9a1b*, *col10a1a*, *col11a1b*, *col12a1a*, *dcn*, *thbs2a*, *thbs3b*, *tgfbi*
Degradation of ECM proteins	**Downregulated**: *mmp15a/b*, *ctsk*, *ctsl*.1 and *ctsba/b*, *sh3pxd2b*	**Downregulated**: *mmp2*, *mmp9*, *mmp11b*, *mmp13a*, *adamts5*, *adamts7*, *adamts10*, *adamts15b*	**Downregulated**: *mmp14a*, *adamts16*

## Data Availability

The raw and processed RNA-seq data was deposited in the Gene Expression Omnibus (GEO) database under the accession number GSE294284.

## Supplementary Materials

The following supporting information can be downloaded at: https://www.mdpi.com/article/10.3390/ijms26136069/s1.
