# Supplementary material for: Transcriptomic Profiling of Zebrafish Mutant for cdkl5 Reveals Dysregulated Gene Expression Associated with Neuronal, Muscle, Visual and Skeletal Development
Source: Int J Mol Sci. 2025 Jun 24;26(13):6069. doi: 10.3390/ijms26136069 (PMC12250029; doi:10.3390/ijms26136069)
Supplement: Supplementary file 1 [file ijms-26-06069-s001.zip › Supplementary Table S12.pdf]

Table S12. List of the primers used in this work. Fw: forward primer; Rev: reverse primer.

Gene name	Sequence (5' to 3')
cdkl5	Fw: AGATGAACCGAAGCCTACTGA
	Rev: GGTGTATCCAAGACCGTAAGG
ahsa1a	Fw: CGCTGGATTGTGGAAGAACGA
	Rev: AGAGGCTTCACCATCTATATTACTGAC
col1a1b	Fw: CTGAGCCTCCATATGTTGATACCGT
	Rev: GACTTCTCACTGCCATAAGCCATCTG
ndr2	Fw: GCTCCTGGATCGTGTTCCCT
	Rev: CTCTCCGTTCTCGTAGTACAGCA
rac3a	Fw: ATCGCCTTAGACCACTTTCATACC
	Rev: CGTTCAATAGTGTCCTTGTCATCCC
nts	Fw: CTCCTCACATCAAAGGTAAAGGCGA
	Rev: TCCTCCAGCATCTGAAGAAGAGTCC
mmp9	Fw: CTGAACCCACTGCTCCTCAACC
	Rev: CCGTCCTTGAAGAAGTGAAGCTCC
neb	Fw: TCTTTGACTATGACCCTGCCGA
	Rev: GACTTCTCATCACCCACACCA
kcna4	Fw: AAAGAGACCCGTCAATGTGCCGT
	Rev: CCTCCTTCACAAAGCCCTCATCC
mmp13a	Fw: TGTCAGTGGCAGAGGTGGATGACTC
	Rev: CGCCACCAGGAACAGATTGTAATATTGAG
olfml3b	Fw: CTTGTTCTTTCTGGGTTAGTCGGG
	Rev: CGCCTCCTTGCTCTTGTCCA
β-actin	Fw: GATGCGGAAACTGGCAAAGG
	Rev: GAGGAGGGCAAAGTGGTAAACG
rps18	Fw: AACACGAACATTGATGGAAGACG
	Rev: ATTAGCAAGGACCTGGCTGTATTT
